# The Effectiveness of Convalescent Plasma for the Treatment of Novel Corona Virus Disease 2019: A Systematic Review and Meta-Analysis

**DOI:** 10.3389/fmed.2021.641429

**Published:** 2021-09-27

**Authors:** Huiling Cao, Li Ming, Long Chen, Xingwang Zhu, Yuan Shi

**Affiliations:** ^1^Department of Neonatology, Children's Hospital of Chongqing Medical University, Chongqing, China; ^2^National Clinical Research Center for Child Health and Disorders, Chongqing, China; ^3^Ministry of Education Key Laboratory of Child Development and Disorders, Chongqing, China; ^4^China International Science and Technology Cooperation Base of Child Development and Critical Disorders, Chongqing, China; ^5^Chongqing Key Laboratory of Pediatrics, Chongqing, China; ^6^Department of Cardiology, Children's Hospital of Chongqing Medical University, Chongqing, China; ^7^Department of Pediatrics, Jiulongpo People's Hospital, Chongqing, China

**Keywords:** convalescent plasma, COVID-19, safety and efficacy, mortality, meta-analysis

## Abstract

**Background:** Coronavirus disease 2019 (COVID-19), sweeping across the world, has created a worldwide pandemic. Effective treatments of COVID-19 are extremely urgent.

**Objective:** To analyze the efficacy and safety of convalescent plasma (CCP) on patients with COVID-19.

**Methods:** All the relevant studies were searched from PubMed, EMBASE,Cochrane library, Scopus, Web of Science, CBM, CNKI, Wan fang, VIP, Medrxiv, Biorxiv, and SSRN on July 19, 2021. PICOS criteria were as follows: (P) the study interests were human subjects with the infection of COVID-19; (I) the intervention of interest was CCP; (C) comparator treatments contained placebo, sham therapy, and standard treatment; (O) the primary outcome was mortality rates by the novel coronavirus. The secondary outcomes included the incidence of serious adverse events, the rate of ICU admission and mechanical ventilation (MV); the length of hospital stay; the duration of MV and ICU stay; the antibody levels, inflammatory factor levels, and viral loads. (S) Only randomized controlled trials (RCTs) of CCP were included. Subanalysis, quality assessment, sensitive analysis, and publication bias were conducted by two reviewers independently.

**Results:** Sixteen RCTs were included and enrolled a total of 16,296 participants in this meta-analysis. The pooled data showed that no significant difference was observed in reducing the rate of overall mortality between CCP treatment group and placebo group (OR 0.96; 95% CI 0.90 to 1.03; *p* = 0.30; *I*^2^ = 6%). According to the results of subgroup analysis, severe or critical patients with CCP showed significant difference in reducing the 28-day mortality of compared with placebo (OR 0.58, 95% CI 0.36 to 0.93, *p* = 0.02, *I*^2^ = 0%). CCP groups have a significantly shorter duration of MV compared with the control group (weighted MD −1.00, 95% CI −1.86 to −0.14 d *p* = 0.02, *I*^2^ = 0%). No significant difference was observed in the length of hospital stay, the duration of ICU, and the rate of ICU and MV. There is no conclusive evidence about the safety of CCP.

**Conclusion:** Convalescent plasma can significantly reduce the 28-day mortality of severe or critical COVID-19 patients and the duration of MV. However, more evidence was needed to prove the safety of convalescent plasma.

## Introduction

The world is suffering from the Coronavirus disease 2019 (COVID-19) pandemic that is affecting hundreds of millions of people around the world. According to World Health Organization (WHO) current updates, the COVID-19 pandemic has spread all over the globe, causing 194 million confirmed cases and over 4 million deaths, last followed on July 31, 2021 (1). In the absence of a definitive treatment, multiple supportive care is used for novel coronavirus pneumonia. Although vaccination is the most effective alternative to prevent COVID-19, the vaccine is just a prophylactic approach that is of no use in the confirmed patients. To date, the only effective drug approved was dexamethasone which can be life-saving for seriously ill COVID-19 patients. The FDA, University of Oxford, and WHO strongly recommend corticosteroids as a treatment of severe and critical COVID-19 patients (2–4). The latest meta-analysis shows that remdesivir was not superior to placebo in mortality rate (5). The results from the Solidarity Therapeutics Trial also showed that hydroxychloroquine, ritonavir/lopinavir, and interferon regimens appeared to have little or no effect on mortality on day 28 among hospitalized patients (6). A recent meta-analysis that involved eight RCTs of tocilizumab was also proved to have no survival benefit on 28-day mortality (7).

Convalescent human plasma which contained COVID-19 neutralizing antibodies (NAbs) could be an effective therapy (8). On account of lacking effective medicine, many countries across the world have put forward using plasma as a therapy in COVID-19 patients for this fatal RNA virus. On August 23, 2020, the United States Food and Drug Administration (FDA) has announced that convalescent plasma (CCP) therapy can be used for critically ill COVID-19 patients as an emergency investigational new drug (9).

Many researches have been suggested that CCP can make a positive difference in the treatment of COVID-19 infection (10–13). However, more and more randomized controlled trials (RCTs) have been finished recently and failed to prove the survival benefit and clinical improvement with CCP, compared with the control group (14–16). Up to now, it is controversial about the efficacy of CCP. Hence, it is essential to conduct a systematic review and meta-analysis to evaluate the clinical efficacy and safety of CCP for the COVID-19 patients.

## Materials and Methods

This meta-analysis was conducted on the basis of the preferred reporting items for systematic reviews and meta-analyses (PRISMA) guidelines (17). The study protocol has been registered in the International Prospective Register of Systematic Reviews (PROSPERO) database (ID: CRD42020177511).

### Search Methods

We firstly searched nine databases (PubMed, Cochrane library, EMBASE, Scopus, Web of Science, CBM, China National Knowledge Infrastructure (CNKI), Wan fang, and VIP) and three online Medrxiv/Biorxiv/SSRN databases which published preprint or peer-reviewed journals from inception to September 14, 2020. We conducted an updated search for newly published articles on July 19, 2021. We combined the terms “COVID-19” or “SARS-Cov-2” or “Novel Coronavirus Pneumonia” with “convalescent plasma” or “convalescent serum” or “convalescent blood product” or “immune plasma” or “hyperimmune immunoglobulin” or “H-IVIG,” or “serotherapy” or “serum therapy” or “convalescent sera treatment” as keywords, and “immune, passive” as medical subject headings (MeSH) terms. All search records were imported to Note Express software. Two reviewers (CHL and ML) independently extracted data and assessed trial methodology by thoroughly reading the abstracts and full text of the studies that met our inclusion criteria. In cases of differences of opinion during the study selection process, a third author (CL) was consulted.

### Criteria for Considering Studies

Inclusion criteria: Only RCTs were considered eligible, evaluating the effectiveness and safety of CCP or hyperimmune immunoglobulin in patients with COVID-19. Exclusion criteria: We excluded cohort study, case–control studies, single arm studies, case reports, editorials, and letters. Publications are limited to the English language or Chinese.

Briefly, the study interests were human subjects who were infected with COVID-19. The intervention of interest was CCP. Control treatments included placebo, sham therapy, and standard treatment. The primary outcome was the mortality rate by the novel coronavirus. The secondary outcomes included the incidence of serious adverse events, the rate of ICU admission and mechanical ventilation (MV), the length of hospital stay, duration of MV and ICU stay, the antibody levels, inflammatory factor levels, and viral loads.

### Quality Assessment

We assessed the quality of all included trials based upon reviewing the details in the part of the method section and supplements of the trials. Quality assessment for RCTs was conducted according to Cochrane collaboration tool for assessing the risk of bias (Rob) (18) with the following domains: “Allocation concealment (selection bias),” “Sequence generation (selection bias),” “Blinding of outcome assessment (detection bias),” “Blinding of participants and personnel (performance bias),” “Incomplete outcome data (attrition bias),” “Selective reporting (reporting bias),” and “Any other bias.” Rob of RCTs can be judged with “high,” “unclear,” or “low.” The two authors (ML, CHL) assessed study quality independently and disagreements were resolved by consensus.

### Data Synthesis and Analysis

Odds ratio (OR) was used for dichotomous outcomes and mean difference (MD) for continuous data with 95% confidence intervals (95% CI). *I*^2^ statistic was used to evaluate the impact of heterogeneity on pooled results. If *I*^2^-value was greater than 50%, it indicated substantial heterogeneity. We used fixed-effects models to pool data when heterogeneity was insignificant. On the other side, random-effects models were used to pool data when significant heterogeneity was identified. Publication bias was assessed by funnel plots and Egger's test. If the funnel plots are asymmetric and have a *P* < 0.05, it indicates the existence of publication bias. Furthermore, sensitivity analysis was done by adjusting the effects of models to assess the robustness of the results. Additionally, subgroup analysis according to the severity of the disease, the infusion time, the volume of the CCP, and the age was performed, respectively. *P* < 0.05 was considered significant. All analyses were performed by using the Review Manager (RevMan) version 5.3 (Copenhagen: The Nordic Cochrane Centre, The Cochrane Collaboration, 2014) and STATA software (version 12.0; StataCorp LP, College Station, TX, USA).

## Results

### Study Inclusion and Characteristics

**Figure 1:**
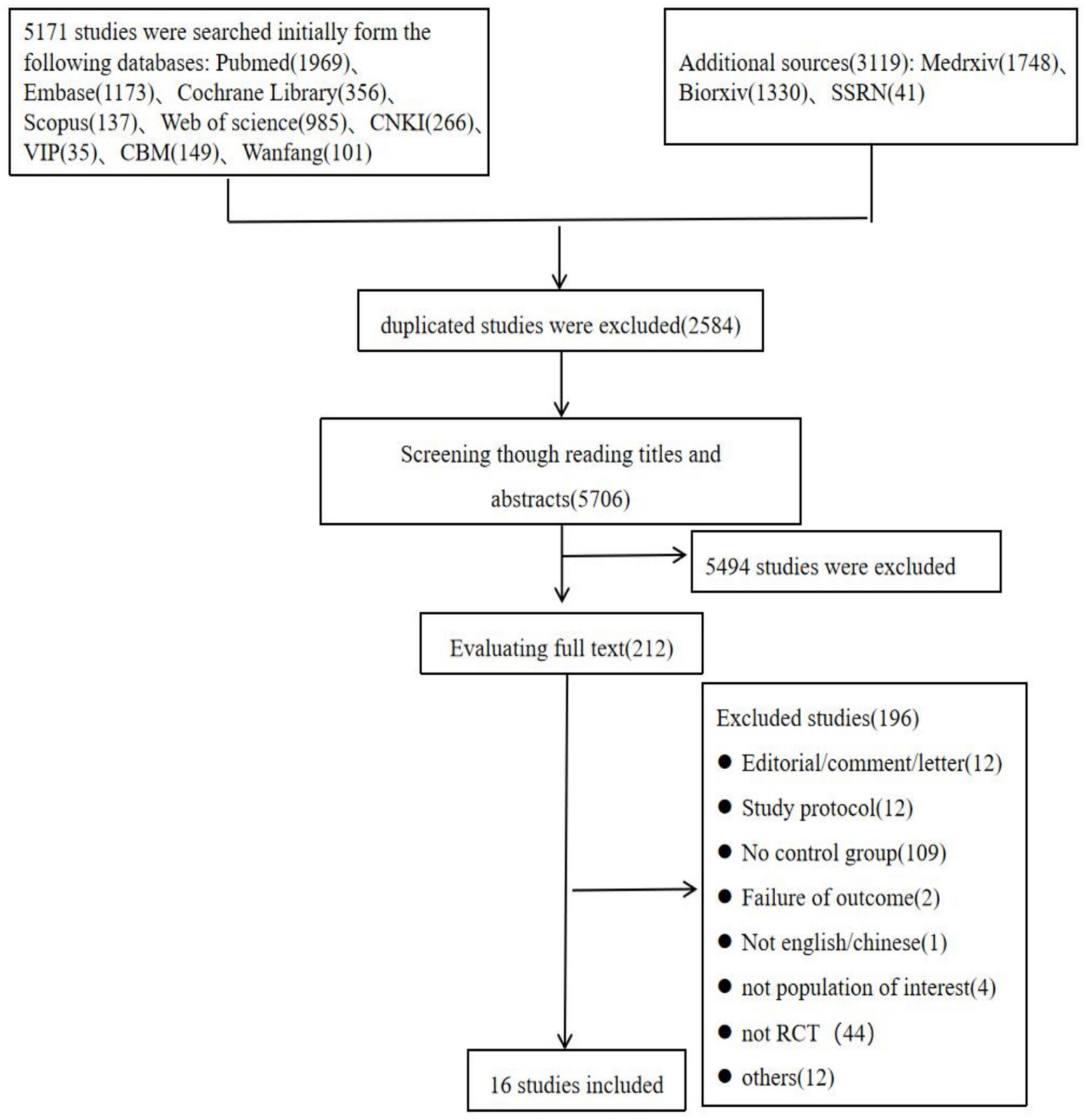
Flow diagram of trial identification and selection.

The search process yielded 8,290 records. After removing duplicates, we screened 5,706 records for this review based upon their titles and abstracts. Of these, we excluded 5,494 studies that did not meet our eligibility criteria. Finally, we evaluated the remaining 212 records and screened the full texts. In this update, we excluded 44 cohort studies or case-control studies. Ultimately, 16 RCTs were included in our review (Figure 1).

We included 16 RCTs (involving the data of RECOVERY, CONCOR-1, and REMAP-CAP), enrolling a total of 16,296 participants in this meta-analysis, of whom 8,526 received CCP, 10 studies concentrated on the severe or critical patients (14, 15, 19–26). Agarwal et al. (27) the population of interest of one study is moderate COVID-19 patients, and one study (28) conducted by Romina Libster focused on mildly ill-infected seniors. Other four studies, which involved the patients with confirmed COVID-19, contained patients with different disease severity (16, 29–31). Six studies are in preprint (20–22, 25, 26, 29). Characteristics of included trials and the trial results are summarized in Table 1.

**Table 1:** Characteristics of 16 studies that assessed the effect of convalescent plasma in patients with COVID-19.

**No**	**Author, journal, country**	**Study, design**	**Population**	**Treatment (n)**	**Control (n)**	**Days of CP after inclusion**
1	Simonovich et al.; *NEJM*; Argentina and Italiano de Buenos Aires	RCT; CP vs. placebo (normal saline solution)	Severe COVID-19 pneumonia	228	105	NA
2	Li et al.; *JAMA*; China	RCT; CP+ ST vs. ST (standard treatment)	Severe and Life- threatening COVID-19	52	51	NA
3	Agarwal et al.; *BMJ*; India	RCT; CP+SOC vs. SOC (standard of care)	Moderate COVID-19	235	229	NA
4	Avendaño-Solà et al.; *medRxiv*; Spain	RCT; CP+SOC vs. SOC	Confirmed COVID-19	38	43	Day 1 after randomization
5	Gharbharan et al.; *Nature Communication*; Netherlands	RCT; CP+SOC vs. SOC	RT-PCR confirmed SARS-CoV-2	43	43	Day 1 of inclusion
6	Libster et al.; *NEJM*; the state of Buenos Aires	RCT; CP vs. placebo (normal saline 0.9%)	Mildly ill infected seniors	80	80	1–1.5 h after inclusion
7	Ray et al.; *medRxiv*; India	RCT; CP+SOC vs. SOC	Severe COVID-19 patients	40	40	Day 1–2 of inclusion
8	Bajpai et al.; *medRxiv*; India	RCT; CP+SMC vs. FFP (fresh frozen plasma)+SMC	Severe COVID-19 patients.	14	15	NA
9	The REMAP-CAP Investigator et al.; *medRxiv*; UK	RCT; CP vs. control	Critical ill patients	1,084	916	Within 48 h
10	AlQahtani et al.; *Scientific Report*; Bahrain	RCT; CP+SOC vs. SOC	Severe COVID-19 patients.	20	20	Over 24 h
11	Pouladzadeh et al.; *Internal and Emergency Medicine*; Iran	RCT; CP+ST vs. ST	Severe COVID-19 patients	30	30	Day 1 after admission
12	Elliott Bennett-Guerrero et al.; *Critical Care Medicine*; New York	RCT; Convalescent Plasma vs. Standard Plasma	Confirmed COVID-19	59	15	Day 0 after admission
13	RECOVERY Collaborative Group et al.; *Lancet*; UK	RCT, CP + usual care vs. usual care	COVID-19 patients	5,795	5,763	Day at randomized/ following day
14	The CONCOR-1 Study Group; *Medrxiv*; Canada, the United States, and Brazil	RCT;CP vs. ST	(severe patients)	625	313	within 24 h of randomization.
15	O'Donnell et al.; *JCI*; USA and Brazil	RCT; CP vs. normal plasma	Severe COVID-19	150	73	NA
16	Körper et al.; *MedRxiv*; German	RCT; CP vs. standard therapy	Severe COVID-19	53	52	within 1 day after randomization
**No**	**Median time of admission**	**Age**	**Doses and frequency**	**Antibody titer**	**Results (CP vs. Control)**	**Adverse events**
1	8 (5–10)	62.5 (53–72.5)	NA	IgG median 1:3,200 (1:800–1:3,200)	clinical status at 30 days: *p* = 0.396; 95% CI 0.81 (0.50–1.31); The 30-day mortality was 10.96 vs. 11.43% *P* > 0.05; HS: 13 vs. 12 days *P* > 0.05; the rate of ICU: 53.9 vs. 26.8% *P* > 0.05; the rate of MV: 60 vs. 22.9% *P* > 0.05;	Serious event 54 (23.7) vs. 19 (18.1) *P* > 0.05; Infusion-related event 13 (5.7) vs. 2 (1.9) *P* > 0.05
2	27 (22–39)	70 (62–80)	4–13 ml/kg; Median plasma infusion volume was 200 mL (IQR, 200–300 mL); 96% a single dose	Ig G > 1:640	Clinical improvement within 28 days 51.9% (27/52) vs. 43.1% (22/51) *P* = 0.26; In severe 91.3% (21/23) vs. 68.2% (15/22) *P* = 0.03; In Critical patients 20.7% (6/29) vs. 24.1% (7/29) *P* = 0.17; 28-day mortality 15.7 vs. 24.0% *P* = 0.30; discharge by 28 days: 51.0 vs. 36.0% *P* = 0.12; negative conversion rate of viral PCR at 72 h: 87.2 vs. 37.5% *P* < 0.001;	2/52 transfusion-related adverse, one is non-severe allergic transfusion reaction, another one is severe transfusion-associated dyspepsia.
3	8 (6–11)	52 (42–60)	200 ml; two doses	NA	All-cause mortality at 28 days or progression to severe Disease: 44/235 (19) vs. 41/229 (18) 95% CI 1.07 (0.73 to 1.58); Mortality within 28:34/235 (15%) vs. 31/229 (14%) 95% CI 1.04 (0.66 to 1.63); median hospital stay 14 (10–19) vs. 13 (10–18) *P* = 0.2; Shortness of breath on day 7: 140/183 (76) vs. 119/181 (66) 95% CI:1.16 (1.02 to 1.32); Negative conversion of SARS-CoV-2 RNA on day 7:95% CI 1.2 (1.04 to 1.5)	3/235 (1%) were assessed transfusion contribute to death
4	8 days	60.5 (46.0–74.0) /61.3 ± 16.3	250–300 ml, one dose	NA	mechanical ventilation or death at day 15:0/38 (0%) vs. 6/43 (14%) *p* = 0.57; at day 29 0/38 (0%) vs. 7/43 (16.3%), Mortality rates: 0/39 (0%) vs. 4/43 (9.3%) at days 15 and 29 *P* = 0.06;	6/38 vs. 7/43 *P* >0.05; Two CP infusion-related AE
5	9 (7–13)	61 (56–70)	300 ml, 1–2 doses	Neutralizing antibody titers >1:80	No difference in mortality on day 15 6/43 (14%) vs. 11/43 (26%) (*p* = 0.95), hospital stay (*p* = 0.68) or day-15 disease severity (*p* = 0.58) was observed	No plasma related serious adverse events were observed
6	<72 h from initiation of symptoms	77.1 ± 8.6	250 ml	Ig G > 1:1,000	Experienced severe respiratory disease: 13/80 (16.2%) vs. 25/80 (31.2%) *p* = 0.026; time to development of severe COVID-19: 15 (15–15) vs. 15 (8.8–15); *p* = 0.028; Mortality: 2/80 vs. 4/80;	No solicited adverse events were observed
7	4.2 ± 2.21	61.36 ± 12.17	200 ml; two doses	NA	HS:13 vs. 17, *P* = 0.098; survival rate:14/40 (35%) vs. 10/40 (25%); for patients aged <67 years: survival rate:8/23 (34.7) vs. 3/27 (11.1) [*P* = 0.042]; duration of hospital stay: 13 days vs. 17 days (*P* = 0.031)	Transfusion-related adverse effects were reported in none of the patients in CPT arm
8	NA	48.1 ± 9.1	250 ml; two doses	Neutralizing antibodies>1:80	Median ICU stay: 5 (4, 5.7) vs. 5 (4, 7) *p* = 0.72; Mean HS: 12.1 ± 4.1 vs. 16.1 ± 5.6 *P* = 0.08; Mortality till 28 days: 3 (21.4%) vs. 1 (6.7%) *P* = 0.33	Transfusion reactions: 1 (7.1%) vs. 1 (6.7%) *P* = 1
9	1.8 (1.0 – 3.2)	60.2 (12.7)	550 ± 150 ml	NA	In-hospital mortality: 37.3% (401/1,075) vs. 38.4% (347/904); The median organ support-free days up to day 21:0 (−1 to 16) vs. 3 (−1 to 16);	There were 44/1,980 (2.2%) participants ≥1 serious adverse event, CP group vs. SOC 32/1,075 (3.0%) vs. 12/905 (1.3%). Patients with ≥ 1 venous thromboembolic event at 90-days, 74/1,075 (6.9) vs. 61/905 (6.7).
10	NA	52.6 (14.9)	2*200 ml	NA	Time on ventilation: (8.25 ± 4.42 days) vs. (10.5 ± 2.9) days (exact *p* = 0.809). Length of stay: 14.1 ± 1.24 days vs. 18.05 ± 2.22 days (*p* = 0.12).Death rate: 2(10%)vs. 1(5%),P=0.55	Two patients treated with plasma reported adverse events during the study that were not considered to be related to therapy
11	NA	53.5 ± 10.3	500 ml (maybe plus)	NA	Death rate: 3 (10%) vs. 5 (16.7%) (*P* = 0.44); LOS:8.66 ± 3.94 vs. 6.66 ± 4.30 (*P* = 0.06); But the WHO severity scores remarkably improved (*p* = 0.01) and the the mean levels of lymphocytes and IL-10 significantly increased while the levels of IL-6, TNF-α, and IFN-γ decreased (*p* < 0.05).	CP therapy had not any serious side effects on patients.
12	9 (6–18)	67 ± 15.8	2 U (total volume approximately 480 mL)	NA	Ventilator-free days through 28 days: median (interquartile range):28 (2–28) vs. 28 (0–28; *p* = 0.86); ≥2 point improvement in the WHO scale:20 vs. 20% (*p* = 0.99). All-cause mortality through 90 days: (27 vs. 33%; *p* = 0.63).	Any SAE in the first 28 d:16 (30) vs. 4 (27)
13	9 (6–12)	63.6 ± 14.7	275 ± 75 ml (2 Units)	S/CO ratio of 6.0 (≥1:100)	28-day mortality:1,398/5,795 (24%) vs. 1,408/5,763 (24%) [*P* = 0.93]; Median HS: 11 vs. 11; Discharged from hospital within 28 days:3,850 (66%) vs. 3,846 (67%) [*P* = 0.5]; Invasive mechanical ventilation:670/5,493 (12%) vs. 681/5,448 (13%) *P* = 0.63	severe allergic reactions:16 vs. 2; There were 13 serious adverse reactions reported to SHOT: 9 patients with pulmonary reactions(including 3 deaths possibly related to transfusion), and 4 patients with serious febrile, 332 allergic or hypotensive reactions (all recovered).
14	8.0 ± 3.8; 8 (5, 10)	67.7 ± 16.0; 69 (58,80)	500 ml	Viral neutralizing antibodies at a titer of >1:160 or antibodies against the (RBD) of the SARS-CoV-2 Spike protein at a titer of >1:100.	Intubation or death occurred at day 30:199/614 (32.4%) vs. 86/307 (28.0%) *P* = 0.18; Length of stay in ICU by day 30: 4.3 ± 7.9 vs. 3.7 ± 7.1 (*P* = 0.22); Serious adverse event by day 30:205 (33.4) vs. 81 (26.4) [*P*= 0.03]; In-hospital death by day 90 :156 (25.0) vs. 69 (22.0)(*P* = 0.33)	serious adverse events (33.4 vs. 26.4%; *RR* = 1.27, 95% CI 1.02–1.57, *p* = 0.034)
15	9	61	NA	1:160 (IQR 1:80–1:320)	Time to clinical improvement:5 (4–6) vs. 7 (5–8) [*P* = 0.231]; day mortality:19 (12.6) vs. 18 (24.6) [*P* = 0.034] Time to hospital discharge: 9 (6–28) vs. 8 (6–22) [*P* = 0.756]; At 28 days, no significant improvement in the clinical scale was observed	Serious adverse events occurred in 39 of 147 (26.5%) vs. 26 of 72 (36.1%); transfusion-associated were reported in 4 of 147 (2.7%) vs. 3 of 72 (4.2%)
16	7 (2–9)	59 (53–65)	837 ml (738–872 ml)	NA	The median time to clinical improvement was 26 days [IQR 15- (n.r.)] vs. 66 days (IQR 13-n.r.) (*p* =0.27). Median time to discharge from hospital was 31 days (IQR 16-n.r.) vs. 51 days (IQR 20–n.r.) (*p* = 0.24).	SAE:22 (41.5) vs. 25 (48.1)

*NA, not available; SAE, serious adverse effects; WHO, world health Organization; CP, convalescent plasma; ICU, intensive care unit; MV, mechanic ventilation; ST, standard treatment; SOC, standard of care; RCT, randomized control trail; FFP, fresh frozen plasma*.

### Rob of Included Studies

**Figure 2:**
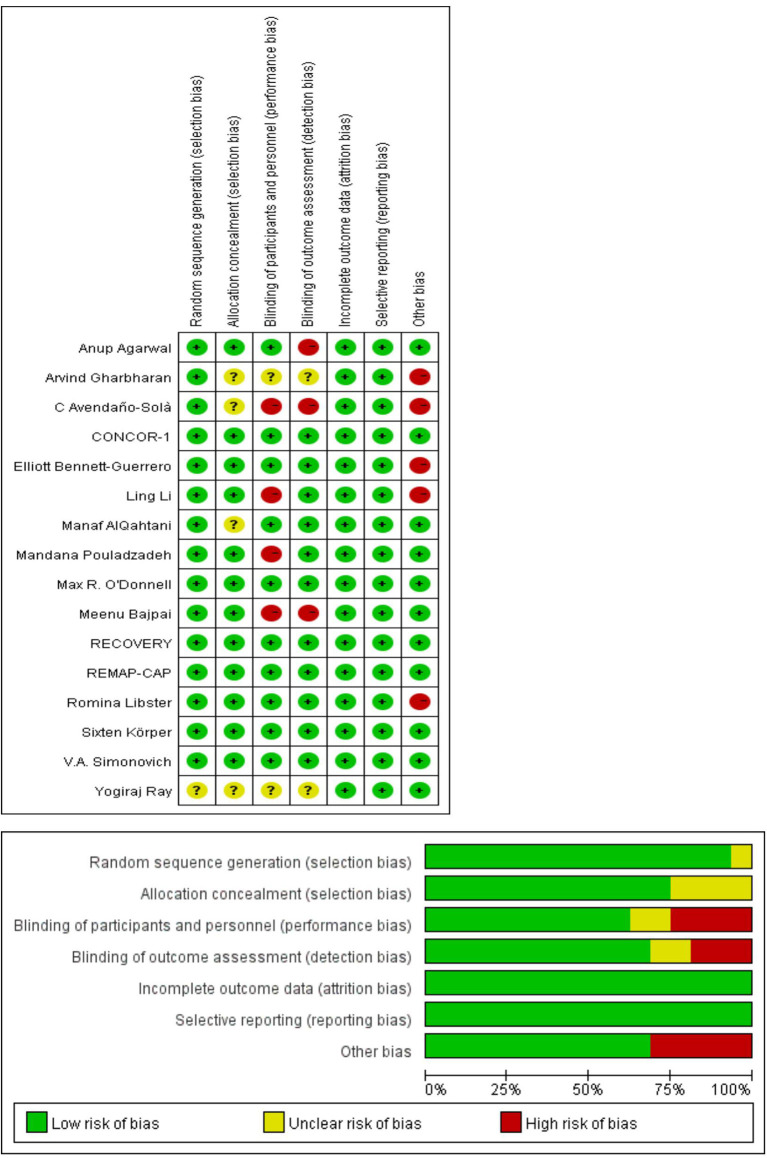
Quality assessment of RCTs.

“Risk of bias” table was done for each included RCTs using the “Risk of bias” tool in the review manager web. The RCTs included were all assessed to be at low Rob in the aspect of attribution bias, random sequence generation, and reporting bias with the exception of one trial (20), in which random sequence generation was deemed unclear. The trials of Ray et al. (20) and Gharbharan et al. (30) were an open-label study, resulting in the unclear risk of allocation concealment, detection bias, and performance bias. The trials of Li et al. (14), Libster et al. (28), Avendaño-Solàe et al. (29), Gharbharan et al. (30), and Bennett-Guerrero et al. (31) were found to be at high risks in other bias because these trials were halted prematurely. Detailed information can be found in Figure 2.

## Primary Outcome of Mortality

**Figure 3:**
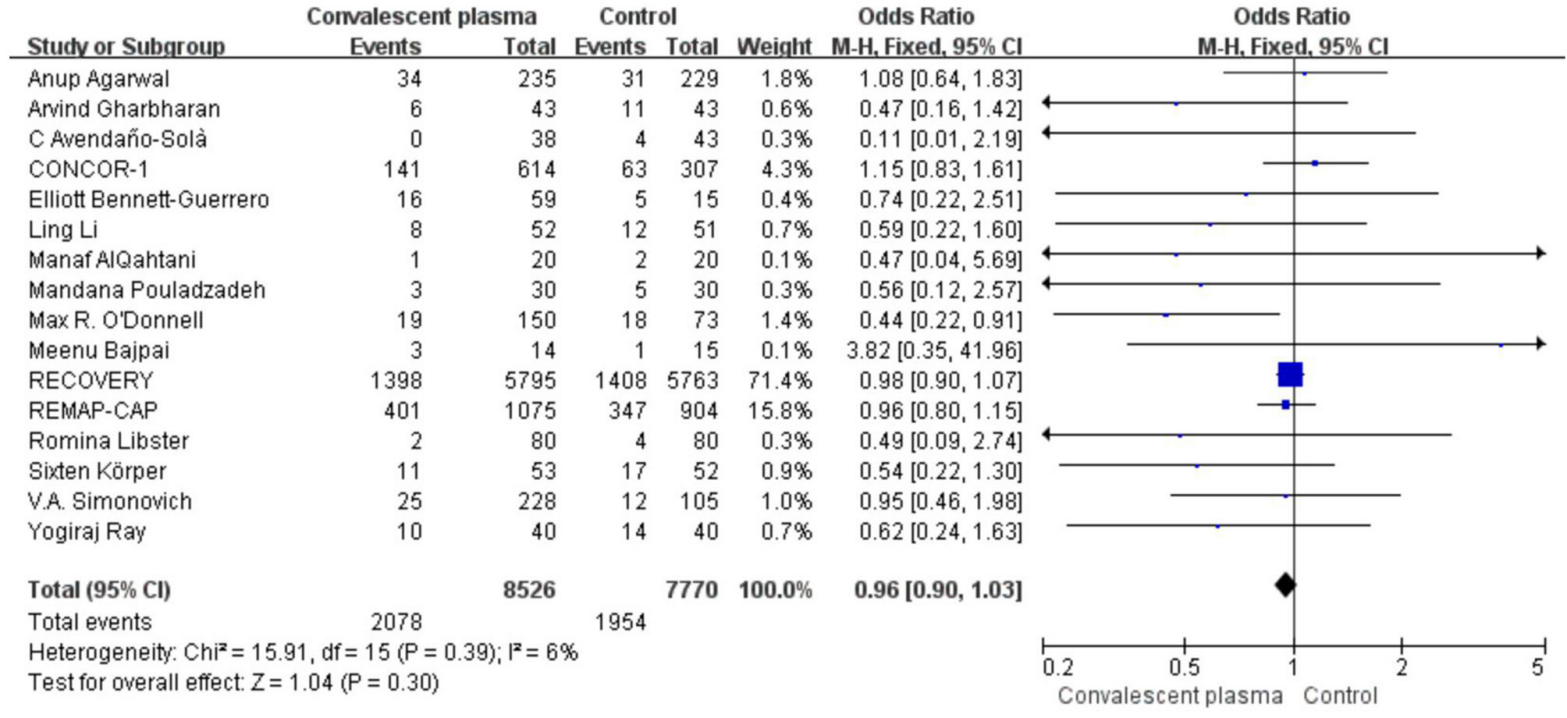
Forest plot of pooled odds ratios (ORs) of mortality among patients treated with convalescent blood products and controls (*n* = 16 studies). Weights are from fixed-effects analysis. CI, confidence interval.

**Figure 4:**
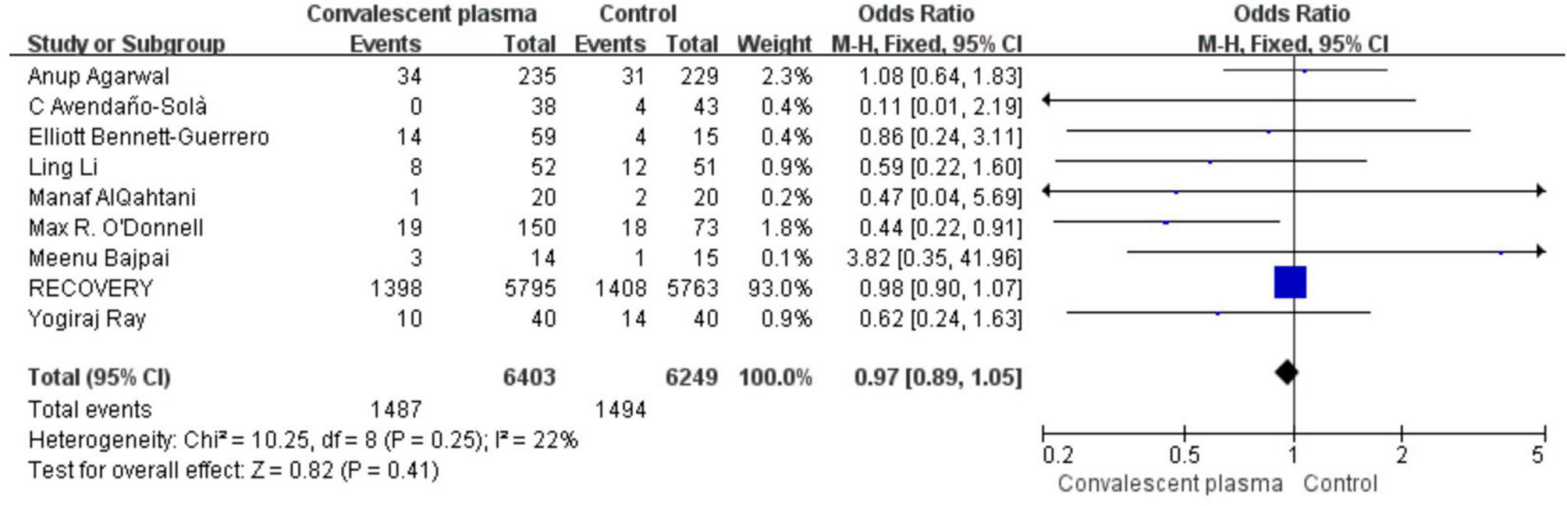
Forest plot of pooled odds ratios (ORs) of mortality on 28-days (*n* = 9 studies). Weights are from fixed-effects analysis. CI, confidence interval.

**Figure 5:**
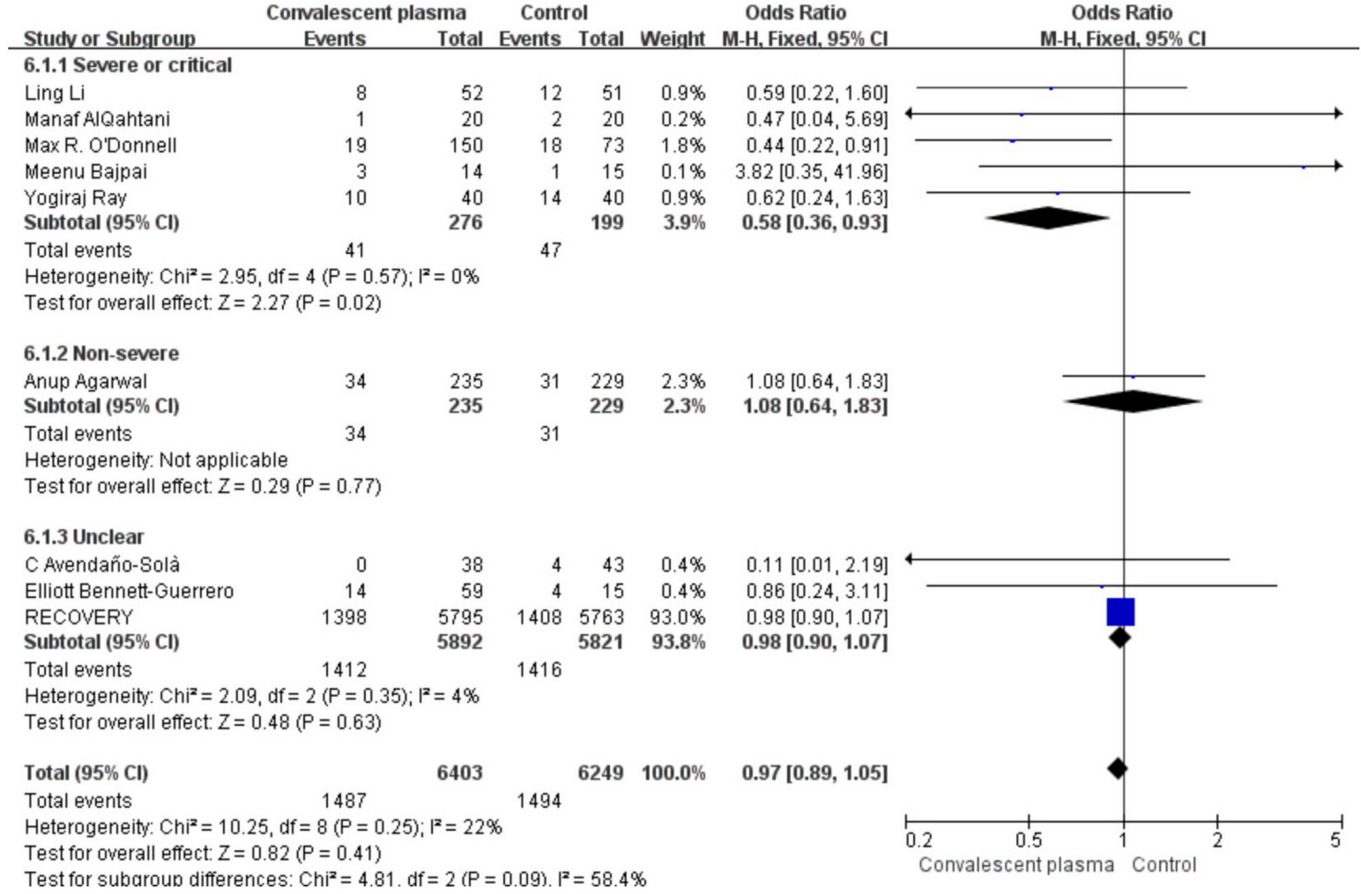
Sub-analysis of pooled odds ratios (ORs) in mortality of 28-days according to severity. Weights are from fixed-effects analysis. CI, confidence interval.

Mortality was assessed in all 16 RCTs. The pooled data (*n* = 16,296) showed that no survival beneficial was observed in the CCP group compared with placebo in patients with COVID-19 (OR 0.96; 95% CI 0.90–1.03; *p* = 0.30; *I*^2^ = 6%; Figure 3). In order to explore the optimal dose, optimal time of infusion, the relationship between mortality and median age, and severity of the disease, a subanalysis was performed. In subgroup analysis, no significant difference between CCP and control group was observed in the severe or critical patients subgroup (OR 0.92, 95% CI 0.80–1.07, *P* = 0.29), infusion time within 3 days of symptom onset subgroup (OR 0.95, 95% CI 0.79–1.14, *P* = 0.56), infusion within 7 days subgroup (OR −0.02, 95% CI −0.06–0.02, *P* = 0.31), age below 65 years subgroup (OR 0.96, 95% CI 0.89–1.03, *P* = 0.27), volume of infusion beyond 500 ml subgroup (OR 0.98, 95% CI 0.84–1.14, *P* = 0.79), and volume of infusion below 300 ml subgroup (OR 0.97, 95% CI 0.90–1.06, *P* = 0.55). Detailed information about subgroups can be found in Supplementary Figures 3A–F. Additionally, we pooled estimates from nine trials, focused on mortality on 28 days, indicating that treatment with CP was not associated with a reduction of mortality (OR 0.97, 95% CI 0.89–1.05, *P* = 0.41, *I*^2^ = 22%; Figure 4). Then we conducted the subanalysis. The studies are layered by the severity of the disease, and the evidence from five trials (*n* =475), which concentrated on severely ill patients with CCP, showed a significant difference in reducing mortality compared with placebo (OR 0.58, 95% CI 0.36–0.93, *P* = 0.02, *I*^2^ = 0%). However, significant difference was not found in nonsevere patients (OR 1.08, 95% CI 0.64–1.83, *P* = 0.77) and unclear group (OR 0.98, 95% CI 0.90–1.07, *P* = 0.63; Figure 5). No differences were founded in the remaining subgroups.

## Secondary Outcomes

### Hospital/ICU/MV Stay

**Figure 6:**
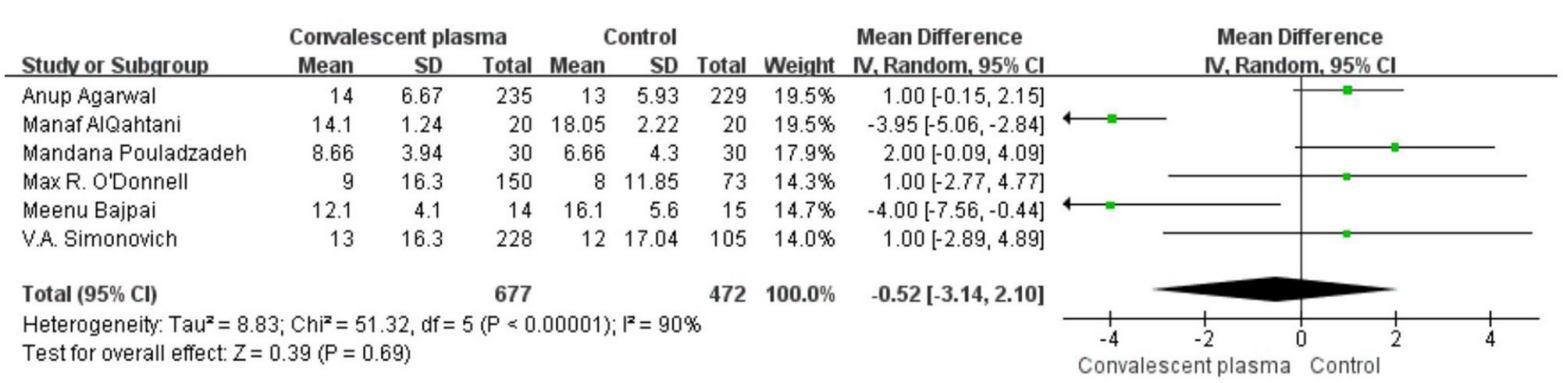
Forest plot of mean difference (MD) of hospital stay among patients treated with convalescent blood products and controls (*n* = 6 studies). Weights are from fixed-effects analysis. CI, confidence interval.

**Figure 7:**
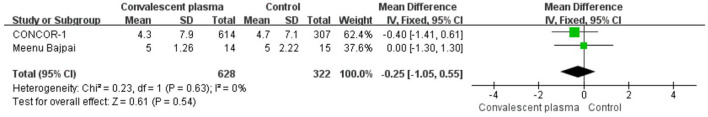
Forest plot of mean difference (MD) of ICU stay among patients treated with convalescent blood products and controls (*n* = 2 studies). Weights are from fixed-effects analysis. CI, confidence interval; ICU, intensive care unit.

**Figure 8:**
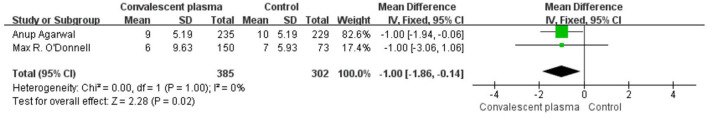
Forest plot of mean difference (MD) of the MV stay among patients treated with convalescent blood products and controls (*n* = 2 studies). Weights are from fixed-effects analysis. CI, confidence interval; MV, mechanic ventilation.

Six studies reported sufficient data to compare the length of hospital stay with treatment and suggested no significant difference of CCP in decreasing length of hospital stay (weighted MD −0.52, 95% CI −3.14–2.10, d; Figure 6). In addition, only 2 trials reported the days of ICU stay (weighted MD −0.25, 95% CI −1.05–0.55, d) was similar between immune plasma treatment and standard care alone (Figure 7). However, there was a significantly shorter duration of MV in CP groups compared with the control group (weighted MD −1.00, 95% CI −1.86 to −0.14d *P* = 0.02, *I*^2^ = 0%; Figure 8).

### The Rate of MV and ICU Admission

**Figure 9:**
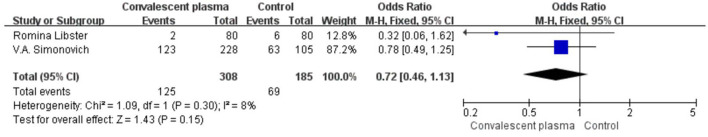
Forest plot of the rate ICU admission among patients treated with convalescent blood products and controls (*n* = 2 studies). Weights are from fixed-effects analysis. CI, confidence interval; ICU, intensive care unit.

**Figure 10:**
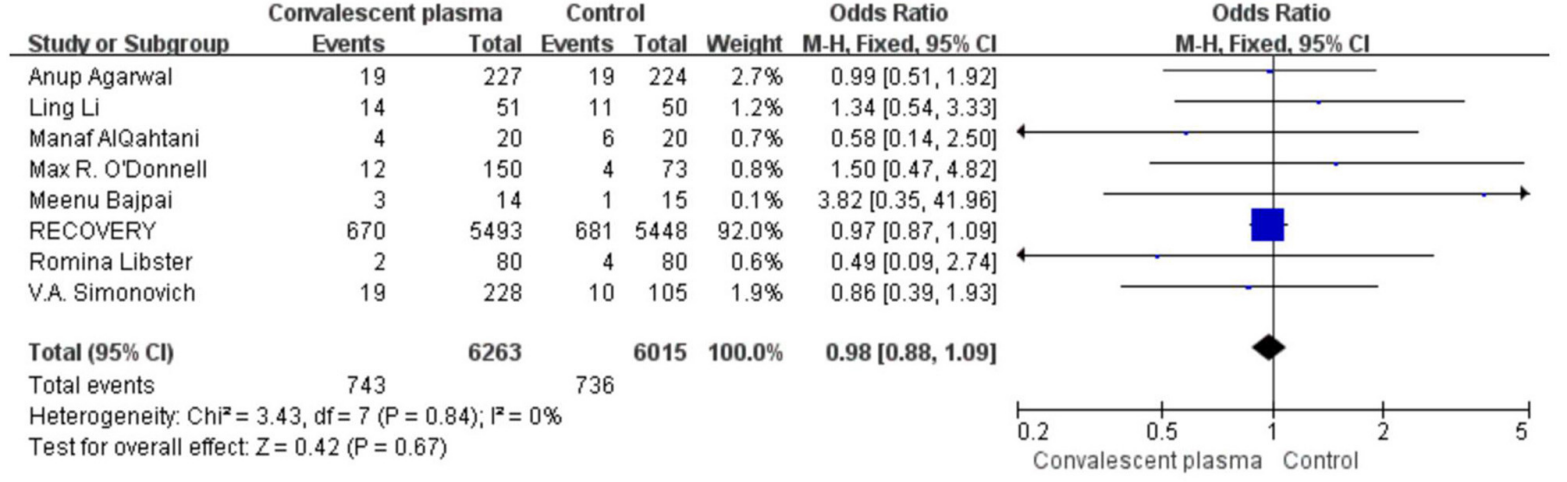
Forest plot of the rate MV among patients treated with convalescent blood products and controls (*n* = 8 studies). Weights are from fixed-effects analysis. CI, confidence interval; MV, mechanic ventilation.

Only two RCTs assessed the rate of ICU admission; pooled data did not show significant differences between the CCP group and the control group (125/308 vs. 69/185, OR 0.72, 95% CI 0.46–1.13, *P* = 0.15; Figure 9). Pooled estimates from eight trials showed that 743/6,263 received invasive MV in the CP group compared with 736/6,015 in the standard care group (OR 0.98; 95% CI 0.88–1.09 *P* = 0.67), suggesting CP cannot significantly decrease the rate of MV (Figure 10).

### Adverse Effect

All studies reported safety outcomes. Four of the 16 studies reported that no transfusion-related adverse effects or any serious side effects were observed in their study. Seven of the 16 RCTs compared adverse effect between CP group and control group and showed no significance. There were four studies reporting on serious adverse events in CCP group without the control group (Table 1). The latest study conducted by the CONCOR-1 Study Group (25) showed that patients of CCP arm have more serious adverse events, compared with patients in the standard of care arm on 30 days (33.4 vs. 26.4%; RR = 1.27, 95% CI 1.02–1.57, *p* = 0.034). Of them, 35 (5.7%) patients in the CCP group were associated with transfusion. The most commonly reported mild adverse event was an allergic reaction, such as chills, rashes, and fever. Serious adverse events are those related to acute lung injury, intravascular volume overload, or serious allergic reactions because of plasma transfusion. Most common events were worsening hypoxemia and respiratory failure. Some experts put forward that CCP should be used with caution because of the hypercoagulability (32). Therefore, one of our studies explored the patients with ≥1 venous thromboembolic event at 90 days, showing no difference between CCP vs. control group [74/1,075 (6.9) vs. 61/905 (6.7)] (22). We cannot conclude that CP can be used safely. Firstly, the time for observation of adverse events was too short and the duration of follow-up across all studies are varied. Secondly, it was difficult to ascertain whether the serious adverse events were related to CCP transfusion or not.

### Other Purposed Outcome

We planned to explore the optimal antibody titer, compare the cytokine levels, and viral loads before and after transfusion. However, different ways of measuring antibody titers and incomplete data on neutralizing antibody titers in CP units limited the search of assessing the relationship between the quality plasma and efficacy. A total of six studies recorded the data of cytokine levels. Four studies just recorded the levels of cytokine at baseline (14, 23, 30, 31). The other two studies compared the cytokine levels before and after transfusion. Their results are inconsistent, one study conducted by Bajpai et al. (21) showed that IL-6 and IL-10 levels were reduced after infusion CCP, whereas IL-6 and IL-10 showed an increase in fresh frozen plasma group. Differences did not attain statistical significance. Another study (24) showed that the mean levels of lymphocytes and IL-10 significantly increased whereas the levels of IL-6, TNF-α, and IFN-γ decreased (*p* < 0.05) in the CCP group when compared with controls. The lack of laboratory data and the difference cytokine level on baseline limited our discussion about the validity of CCP. Just one study compared the viral load before and after transfusion and indicated no difference between the two groups.

### Publication Bias

We evaluated publication bias of the outcome of the rate of overall mortality, rate of mortality on 28 days, hospital stay, the rate of MV, the rate of ICU, the length of ICU, and the duration of MV. The results suggested that the funnel plot for all of the outcomes we assessed was symmetrical, and Egger's test was nonsignificant with the exception of the rate of overall mortality, suggesting the results of mortality need to be cautious. The details can be found in Supplementary Figures 3G,H, 4B,C, 5A,B, 6A,B, 7A,B, 8A,B, 9A,B.

### Sensitive Analysis

When different effects models (Fixed-effects models or random-effects models) were applied, we found that the outcome of the hospital day was changed, suggesting that the consequence was not robustness and the results need to be cautious. The details are presented in Table 2.

**TABLE 2:** The sensitive analysis.

**Outcome**	**Effect index**	**Random-effects models**			**Fixed-effects models**		
		**Effects**	**95% CI**	***P*-value**	**Effects**	**95% CI**	***P*-value**
Mortality	OR	0.95	(0.86,1.05)	*p* = 0.29	0.96	(0.90,1.03)	*P* = 0.30
Mortality of 28 day	OR	0.85	(0.65,1.11)	*P* = 0.22	0.97	(0.89,1.05)	*P* = 0.41
HS	MD	−0.52	(−3.14,2.10)	*P* = 0.69	−1.07	(−1.77,-0.36)	*P* = 0.003
Duration of ICU	MD	−0.25	(−1.05,0.55)	*P* = 0.54	−0.25	(−1.05,0.55)	*P* = 0.54
The duration of MV	MD	−1	(−1.86,−0.14)	*P* = 0.02	−1	(−1.86,−0.14)	*P* = 0.02
Rate of ICU	OR	0.71	(0.40,1.23)	*P* = 0.22	0.72	(0.46,1.13)	0.15
The rate of MV	OR	0.98	(0.88,1.09)	*P* = 0.66	0.98	(0.88,1.09)	0.67

*HS, hospital stay; ICU, intensive care unit; MV, mechanic ventilation; OR, odds ratios; MD, mean difference*.

## Discussion

Our analysis suggests that patients with coronavirus disease 2019 who received transfusion with CCP have no significant reduction in the risk for death. Our subanalysis indicates that the reduction of 28-day mortality occurred in severe or critical patients, whereas no difference was observed in nonsevere patients. CP treatment also decreases the duration of MV. Otherwise, we found a nonsignificant reduction in days in the hospital, rate of MV, days in ICU, and the rate of ICU admission. Twelve studies reported severe adverse effects and eight trials reported transfusion-specific serious adverse reactions in the CCP group. Due to the ambiguous reasons of serious adverse effects, we cannot judge the safety of CP.

Considering the efficacy of CCP in treating previous viral diseases such as the severe acute respiratory syndrome (SARS), 1918 flu epidemic, and H1N1 influenza (33–36), CCP, as a way of passive immunity, can provide NAbs that restrain the infection by binding to spike1-receptor binding protein (S1-RBD), S1-N-terminal domain, and S2, thus inhibiting virus entry and limiting viral amplification. In addition, there are also other protective antibodies, such as immunoglobulin G (IgG) and immunoglobulin M(IgM) which contribute to recovery improvement (37). Gazzaruso et al. (38) provided that the beneficial impact of mortality of CP may contribute to the antithrombin. Some researchers have put forward that CP therapy can be a potential therapy in COVID-19 (8). However, the effectiveness of CP is controversial with more and more studies conducted. Our study was based on the analysis of 16 RCTs. We retrieved the newest RCTs to provide a solid evidence. We found that CCP was associated with a shorter duration of MV compared with the control group. Few research discusses the duration of MV. Only two of the 16 RCTs (15, 27) studies have reported the duration of MV, and so the results need to be verified by more RCTs studies. In this meta-analysis, we find no beneficial effects of CP in overall mortality, the rate of MV or ICU admission, and the length of hospital stay and duration of ICU compared with the control group. This is consistent with the findings of the previous meta-analysis. The study of Cochrane, which included 12 RCTs, showed the same results. Compared with Cocharane (39), we included four new RCTs containing the results of the REMAP-CAP and the CONCOR-Study group. The researches of the two groups relatively reduced the impact of the large sample study, RECOVERY, to the results. Similarly, the study conducted by Janiaud et al. (40) involving a total of 11,782 patients from 10 RCTs indicated that CP was not associated with a decrease in all-cause mortality or any benefit for other clinical outcomes. The other two meta-analysis showed a reduction in mortality but disappeared in RCTs, strengthening our results (41, 42). Contrary to our study, several previous meta-analyses (43–46) showed that CP could help reduce mortality in COVID-19 patients, and their level of evidence was lower than the present study because most of them included not only RCTs but also observation studies in their analysis. Therefore, our study provided more solid evidence. A meta-analysis conducted by Luo et al. (43) put forward that patients with severe COVID-19 benefit more from the CCP transfusion; so we conducted the subanalysis and we found that there was a significantly lower 28-day mortality rate in the severe and critical patients treated with CP compared with the control groups (41/276 vs. 47/199, *P* = 0.02, OR 0.58 95% CI 0.36–0.93). An observational prospective study (*n* = 2,432) also offers an evidence that CP group has a lower mortality than the nontransfused group in the critical cases (44.3 vs. 48.9%) (47). Our analysis should be interpreted with caution as only one of the five RCTs showed a reduction in 28-day mortality, but the sample size of this study accounted for 46% of the weight in the meta-analysis, suggesting the results were largely dominated by this trial. An RCT conducted by Libster et al. (28) had demonstrated that early administration of plasma (1–1.5 h after inclusion) with high titers of antibody (IgG titers above a median of 1:3,200) against SARS-CoV2 to infected elderly (mean age 77.1) reduced progression to severe COVID-19 by 48%. Livia Hegerova et al. (48) explored the relationship between death and infusion time, and they found that CP given prior to 7 days hospitalization can reduce the mortality at the 14 day follow-up (0 vs. 25%). Salazar et al. (49) also suggested that a significant mortality reduction in patients, specifically in patients transfused within 72 h of admission with plasma. Another two studies (44, 50) provide the evidence that earlier administration of plasma within the clinical course of COVID-19 is more likely to reduce mortality. In our study, we divided the studies according to the time of infusion. Results showed that there is no beneficial in mortality, no matter less than 7 or 3 days (Supplementary Figures 3B,C). We think it is associated with the two large sample researches (RECOVERY and REMAP-CAP).They weigh too much that they can cover other studies. Study conducted by Romina Libster et al. explored the mortality in elderly patients (mean age 77.1) and showed a reduction in the rate of experienced severe respiratory disease, [13/80 (16.2%) vs. 25/80 (31.2%) *p* = 0.026]. A cohort study conducted by Yoon et al. (51) showed CCP recipients <65 years had four-fold lower mortality. However, another cohort study (48) provided there was a significant increase in the rate of hospital discharge among patients 65-years-old or greater who received CP (RR 1.86, 95% CI 1.03–3.36). We did the subanalysis, but we did not find any difference between the CP group and the control group (Supplementary Figure 3D). We also explored the optimal infusion volume. Regretful, we did not find any difference significant. In our meta-analysis, there were eight trials of reported transfusion-associated severe adverse events. Recently, Sanfilippo et al. (32) put forward that transfusion of CP has a high risk of pulmonary embolism, as plasma contains procoagulant factors. Still, some researches also provided other adverse effects, such as the antibody-dependent enhancement and a high risk of an HIV epidemic in low- and middle-income nations (52, 53). However, according to the results of Expanded Access Program which contains 20,000 Hospitalized Patients the rate of serious adverse events within 4 h of transfusion was less than 1%, thrombotic events or thromboembolic (*n* = 113; <1%) (54). The REMAP-CAP (22) Investigator followed up to 90 days to explore the rate of thromboembolic events and found no significant difference between CP and control group [74/1,075 (6.9) vs. 61/905 (6.7)]. The RECOVERY (16) also observed no significant differences in the frequency of sudden worsening in respiratory status, temperature rise, sudden hypotension, clinical hemolysis, thrombotic events, and cardiac arrhythmia. Conversely, another large sample study, CONCOR-1, found more serious adverse events in the CCP group than the control group (33.4 vs. 26.4%; RR = 1.27, 95% CI 1.02–1.57, *p* = 0.034) (25); however, the group did not analyze whether it is associated with a transfusion or not. We cannot get a conclusion that CCP is a safe way due to the limited data and inconsistent standards. We look forward to more and more studies to explore the side effects about CP. Simultaneously, It would be great to have a standard of infusion-associated side effects.

## Limitation

Our findings are provocative, but our meta-analysis has important limitations. The gap of the sample is too large, and the results of three large sample studies will obscure the results of other studies, especially the study of RECOVERY. Some of the studies were low quality, with a moderate or high Rob. We were not able to pool all data reported for outcomes regarding the neutralization antibodies, cytokine level, viral load, side-effect of transfusion due to variability in the measuring and incomplete reporting of these outcomes.

## Conclusion

In conclusion, despite some limitations, this meta-analysis demonstrates that CCP can significantly reduce the 28-day mortality of severe or critical COVID-19 patients and the duration of MV. No significant difference was observed in the length of hospital stay, the duration of ICU, the rate of ICU, and MV. However, more evidence was needed to prove the safety of CCP.

## Data Availability Statement

The original contributions presented in the study are included in the article/Supplementary Material, further inquiries can be directed to the corresponding author/s.

## Ethics Statement

Ethical review and approval was not required for the study on human participants in accordance with the local legislation and institutional requirements. Written informed consent from the participants' legal guardian/next of kin was not required to participate in this study in accordance with the national legislation and the institutional requirements.

## Author Contributions

HC and YS conceived and designed the study. LM and HC collected the data. LM and LC analyzed the data. HC wrote the first manuscript draft. XZ and YS revised it critically for important intellectual content. YS is the guarantor. All the authors provided intellectual input, had access to the complete dataset, contributed to manuscript revisions, and approved of the final version.

## Conflict of Interest

The authors declare that the research was conducted in the absence of any commercial or financial relationships that could be construed as a potential conflict of interest.

## Publisher's Note

All claims expressed in this article are solely those of the authors and do not necessarily represent those of their affiliated organizations, or those of the publisher, the editors and the reviewers. Any product that may be evaluated in this article, or claim that may be made by its manufacturer, is not guaranteed or endorsed by the publisher.
