# Supplementary material for: The Effectiveness of Convalescent Plasma for the Treatment of Novel Corona Virus Disease 2019: A Systematic Review and Meta-Analysis
Source: Front Med (Lausanne). 2021 Sep 27;8:641429. doi: 10.3389/fmed.2021.641429 (PMC8502818; doi:10.3389/fmed.2021.641429)

Figure 3A Sub-analysis of pooled odds ratios (ORs) in mortality according to severity

Study or Subgroup	Convalescent plasma Events	Convalescent plasma Total	Control Events	Control Total	Weight	Odds Ratio M-H, Fixed, 95% CI
**4.1.1 Severe or critical**						
CONCOR-1	141	614	63	307	4.3%	1.15 [0.83, 1.61]
Ling Li	8	52	12	51	0.7%	0.59 [0.22, 1.60]
Manaf AlQahtani	1	20	2	20	0.1%	0.47 [0.04, 5.69]
Mandana Pouladzadeh	3	30	5	30	0.3%	0.56 [0.12, 2.57]
Max R. O'Donnell	19	150	18	73	1.4%	0.44 [0.22, 0.91]
Meenu Bajpai	3	14	1	15	0.1%	3.82 [0.35, 41.96]
REMAP-CAP	401	1075	347	904	15.8%	0.96 [0.80, 1.15]
Sixten Körper	11	53	17	52	0.9%	0.54 [0.22, 1.30]
V.A. Simonovich	25	228	12	105	1.0%	0.95 [0.46, 1.98]
Yogiraj Ray	10	40	14	40	0.7%	0.62 [0.24, 1.63]
**Subtotal (95% CI)**		**2276**		**1597**	**25.2%**	**0.92 [0.80, 1.07]**
Total events	622		491			
Heterogeneity: $Chi^2 = 10.78$, df = 9 (P = 0.29); $I^2 = 17\%$						
Test for overall effect: Z = 1.05 (P = 0.29)						
**4.1.2 Non-severe**						
Anup Agarwal	34	235	31	229	1.8%	1.08 [0.64, 1.83]
Romina Libster	2	80	4	80	0.3%	0.49 [0.09, 2.74]
**Subtotal (95% CI)**		**315**		**309**	**2.1%**	**1.01 [0.61, 1.66]**
Total events	36		35			
Heterogeneity: $Chi^2 = 0.75$, df = 1 (P = 0.39); $I^2 = 0\%$						
Test for overall effect: Z = 0.02 (P = 0.98)						
**4.1.3 Unclear**						
Arvind Gharbharan	6	43	11	43	0.6%	0.47 [0.16, 1.42]
C Avendaño-Solà	0	38	4	43	0.3%	0.11 [0.01, 2.19]
Elliott Bennett-Guerrero	16	59	5	15	0.4%	0.74 [0.22, 2.51]
RECOVERY	1398	5795	1408	5763	71.4%	0.98 [0.90, 1.07]
**Subtotal (95% CI)**		**5935**		**5864**	**72.7%**	**0.97 [0.90, 1.06]**
Total events	1420		1428			
Heterogeneity: $Chi^2 = 3.92$, df = 3 (P = 0.27); $I^2 = 24\%$						
Test for overall effect: Z = 0.60 (P = 0.55)						
**Total (95% CI)**		**8526**		**7770**	**100.0%**	**0.96 [0.90, 1.03]**
Total events	2078		1954			
Heterogeneity: $Chi^2 = 15.91$, df = 15 (P = 0.39); $I^2 = 6\%$						
Test for overall effect: Z = 1.04 (P = 0.30)						
Test for subgroup differences: $Chi^2 = 0.40$, df = 2 (P = 0.82), $I^2 = 0\%$						

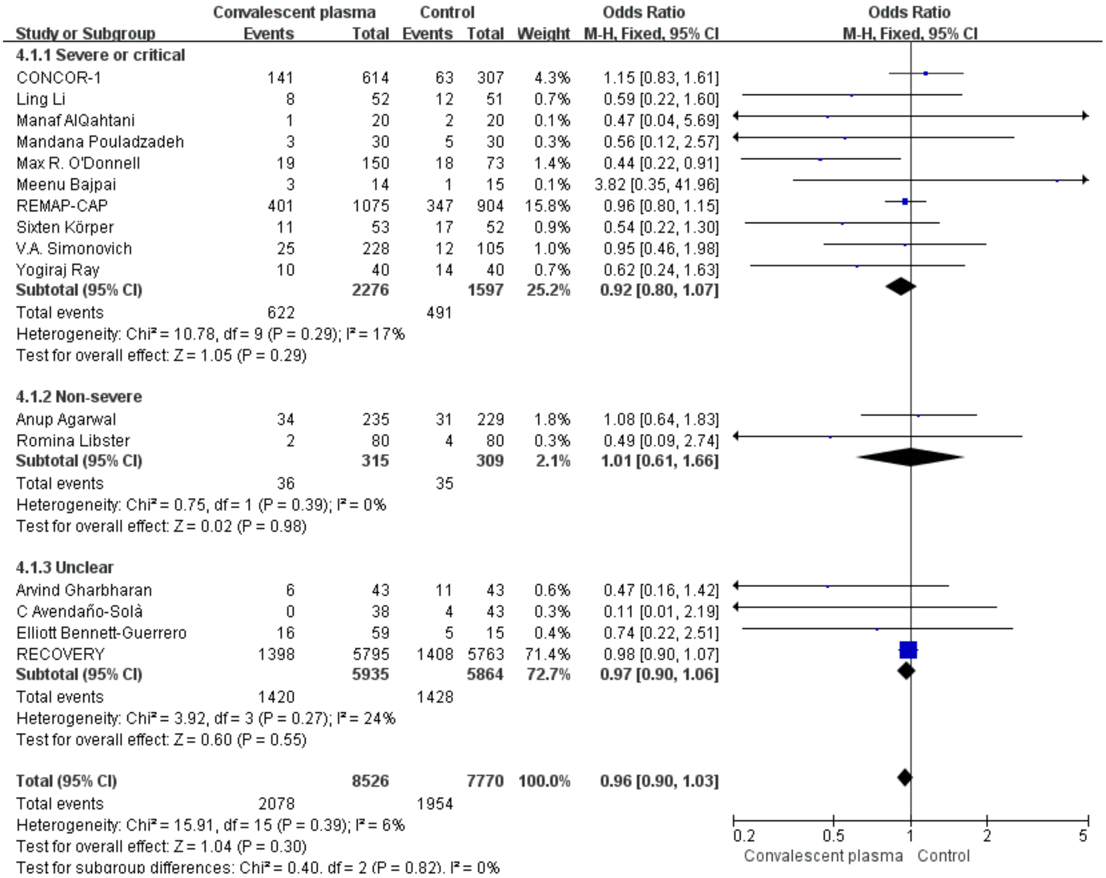

Weights are from fixed-effects analysis. Abbreviation: CI, confidence interval;

Figure 3B Sub-analysis of pooled odds ratios (ORs) in mortality according to infusion time (3 days)

Study or Subgroup	Convalescent plasma Events	Convalescent plasma Total	Control Events	Control Total	Weight	Odds Ratio M-H, Fixed, 95% CI
**30.1.1 >3days**						
Anup Agarwal	34	235	31	229	1.8%	1.08 [0.64, 1.83]
Arvind Gharbharan	6	43	11	43	0.6%	0.47 [0.16, 1.42]
C Avendaño-Solà	0	38	4	43	0.3%	0.11 [0.01, 2.19]
CONCOR-1	141	614	63	307	4.3%	1.15 [0.83, 1.61]
Elliott Bennett-Guerrero	16	59	5	15	0.4%	0.74 [0.22, 2.51]
Ling Li	8	52	12	51	0.7%	0.59 [0.22, 1.60]
Max R. O'Donnell	19	150	18	73	1.4%	0.44 [0.22, 0.91]
RECOVERY	1398	5795	1408	5763	71.4%	0.98 [0.90, 1.07]
Sixten Körper	11	53	17	52	0.9%	0.54 [0.22, 1.30]
V.A. Simonovich	25	228	12	105	1.0%	0.95 [0.46, 1.98]
Yogiraj Ray	10	40	14	40	0.7%	0.62 [0.24, 1.63]
**Subtotal (95% CI)**		**7307**		**6721**	**83.5%**	**0.97 [0.89, 1.05]**
Total events	1668		1595			
Heterogeneity: Chi² = 13.21, df = 10 (P = 0.21); I² = 24%						
Test for overall effect: Z = 0.86 (P = 0.39)						
**30.1.2 ≤3days**						
REMAP-CAP	401	1075	347	904	15.8%	0.96 [0.80, 1.15]
Romina Libster	2	80	4	80	0.3%	0.49 [0.09, 2.74]
**Subtotal (95% CI)**		**1155**		**984**	**16.0%**	**0.95 [0.79, 1.14]**
Total events	403		351			
Heterogeneity: Chi² = 0.58, df = 1 (P = 0.45); I² = 0%						
Test for overall effect: Z = 0.58 (P = 0.56)						
**30.1.3 Unclear**						
Manaf AlQahtani	1	20	2	20	0.1%	0.47 [0.04, 5.69]
Mandana Pouladzadeh	3	30	5	30	0.3%	0.56 [0.12, 2.57]
Meenu Bajpai	3	14	1	15	0.1%	3.82 [0.35, 41.96]
**Subtotal (95% CI)**		**64**		**65**	**0.5%**	**0.88 [0.30, 2.57]**
Total events	7		8			
Heterogeneity: Chi² = 2.03, df = 2 (P = 0.36); I² = 1%						
Test for overall effect: Z = 0.23 (P = 0.81)						
**Total (95% CI)**		**8526**		**7770**	**100.0%**	**0.96 [0.90, 1.03]**
Total events	2078		1954			
Heterogeneity: Chi² = 15.91, df = 15 (P = 0.39); I² = 6%						
Test for overall effect: Z = 1.04 (P = 0.30)						
Test for subgroup differences: Chi² = 0.06, df = 2 (P = 0.97), I² = 0%						

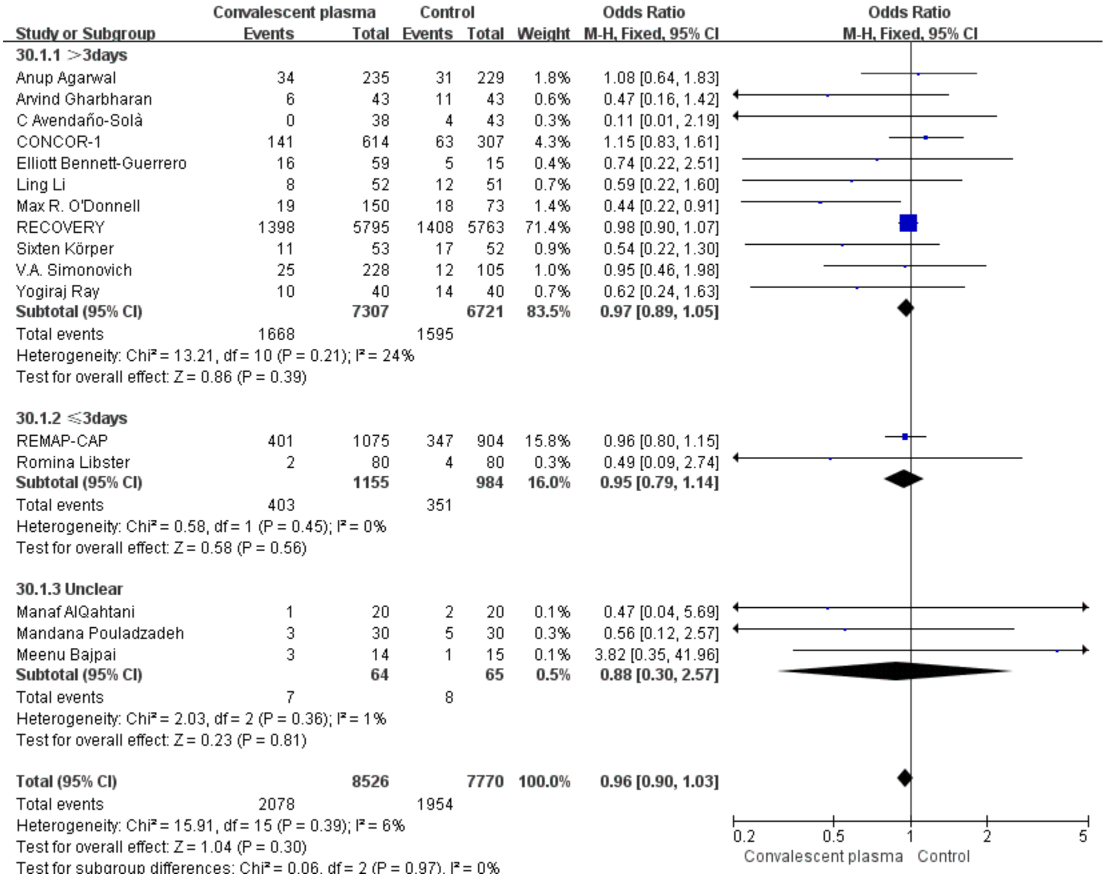

Weights are from fixed-effects analysis. Abbreviation: CI, confidence interval;

Figure 3C Sub-analysis of pooled odds ratios (ORs) in mortality according to infusion time (7 days)

Study or Subgroup	Convalescent plasma Events	Convalescent plasma Total	Control Events	Control Total	Weight	Risk Difference M-H, Fixed, 95% CI
**13.1.1 ≤7days**						
REMAP-CAP	401	1075	347	904	12.2%	-0.01 [-0.05, 0.03]
Romina Libster	2	80	4	80	1.0%	-0.03 [-0.08, 0.03]
Sixten Körper	11	53	17	52	0.7%	-0.12 [-0.29, 0.05]
Yogiraj Ray	10	40	14	40	0.5%	-0.10 [-0.30, 0.10]
**Subtotal (95% CI)**		**1248**		**1076**	**14.4%**	**-0.02 [-0.06, 0.02]**
Total events	424		382			
Heterogeneity: Chi² = 2.17, df = 3 (P = 0.54); I² = 0%						
Test for overall effect: Z = 1.02 (P = 0.31)						
**13.1.2 >7days**						
Anup Agarwal	34	235	31	229	2.9%	0.01 [-0.05, 0.07]
Arvind Gharbharan	6	43	11	43	0.5%	-0.12 [-0.28, 0.05]
C Avendaño-Solà	0	38	4	43	0.5%	-0.09 [-0.19, 0.00]
CONCOR-1	141	614	63	307	5.1%	0.02 [-0.03, 0.08]
Elliott Bennett-Guerrero	16	59	5	15	0.3%	-0.06 [-0.33, 0.20]
Ling Li	8	52	12	51	0.6%	-0.08 [-0.23, 0.07]
Max R. O'Donnell	19	150	18	73	1.2%	-0.12 [-0.23, -0.01]
RECOVERY	1398	5795	1408	5763	71.9%	-0.00 [-0.02, 0.01]
V.A. Simonovich	25	228	12	105	1.8%	-0.00 [-0.08, 0.07]
**Subtotal (95% CI)**		**7214**		**6629**	**84.8%**	**-0.00 [-0.02, 0.01]**
Total events	1647		1564			
Heterogeneity: Chi² = 11.42, df = 8 (P = 0.18); I² = 30%						
Test for overall effect: Z = 0.66 (P = 0.51)						
**13.1.3 Unclear**						
Manaf AlQahtani	1	20	2	20	0.2%	-0.05 [-0.21, 0.11]
Mandana Pouladzadeh	3	30	5	30	0.4%	-0.07 [-0.24, 0.10]
Meenu Bajpai	3	14	1	15	0.2%	0.15 [-0.10, 0.40]
**Subtotal (95% CI)**		**64**		**65**	**0.8%**	**-0.01 [-0.12, 0.10]**
Total events	7		8			
Heterogeneity: Chi² = 2.17, df = 2 (P = 0.34); I² = 8%						
Test for overall effect: Z = 0.24 (P = 0.81)						
**Total (95% CI)**		**8526**		**7770**	**100.0%**	**-0.01 [-0.02, 0.01]**
Total events	2078		1954			
Heterogeneity: Chi² = 16.56, df = 15 (P = 0.35); I² = 9%						
Test for overall effect: Z = 1.04 (P = 0.30)						
Test for subgroup differences: Chi² = 0.54, df = 2 (P = 0.76), I² = 0%						

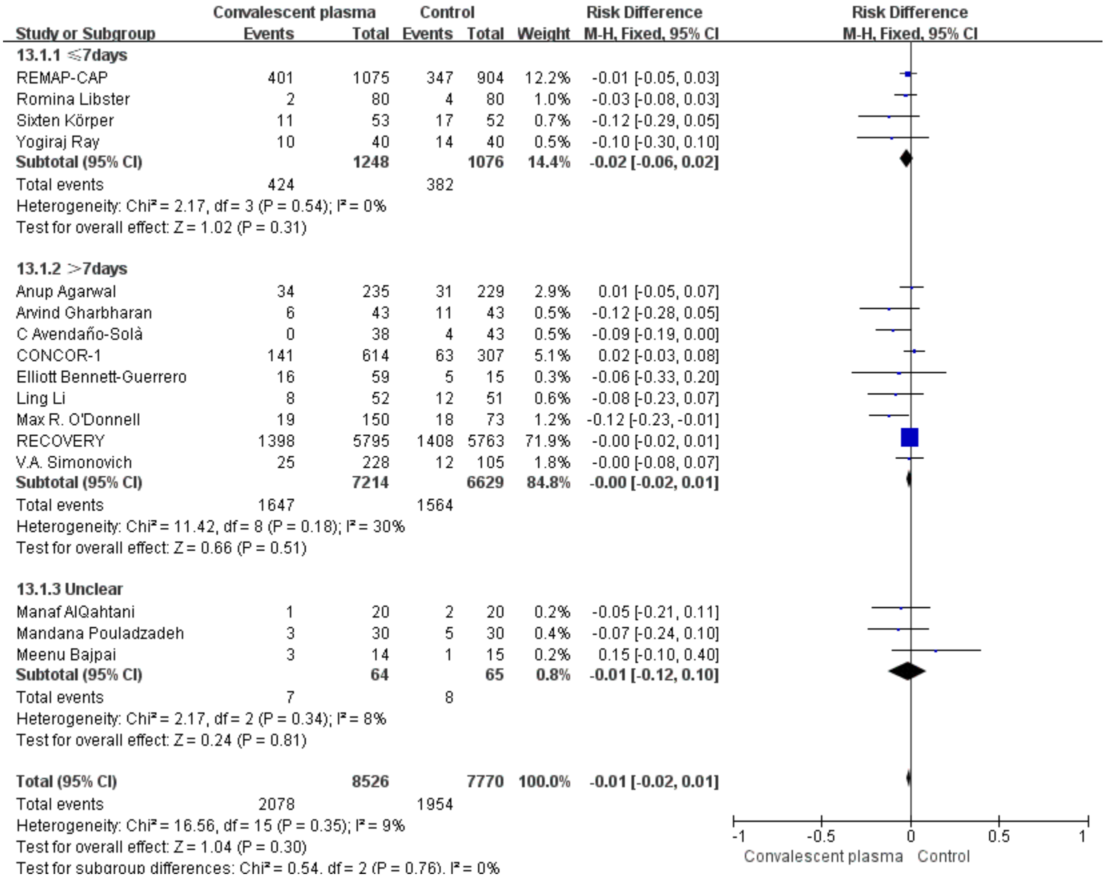

Weights are from fixed-effects analysis. Abbreviation: CI, confidence interval;

Figure 3D Sub-analysis of pooled odds ratios (ORs) in mortality according to age(65 years)

Study or Subgroup	Convalescent plasma Events	Convalescent plasma Total	Control Events	Control Total	Weight	Odds Ratio M-H, Fixed, 95% CI
**16.1.1 ≤65years**						
Anup Agarwal	34	235	31	229	1.8%	1.08 [0.64, 1.83]
Arvind Gharbharan	6	43	11	43	0.6%	0.47 [0.16, 1.42]
C Avendaño-Solà	0	38	4	43	0.3%	0.11 [0.01, 2.19]
Manaf AlQahtani	1	20	2	20	0.1%	0.47 [0.04, 5.69]
Mandana Pouladzadeh	3	30	5	30	0.3%	0.56 [0.12, 2.57]
Max R. O'Donnell	19	150	18	73	1.4%	0.44 [0.22, 0.91]
Meenu Bajpai	3	14	1	15	0.1%	3.82 [0.35, 41.96]
RECOVERY	1398	5795	1408	5763	71.4%	0.98 [0.90, 1.07]
REMAP-CAP	401	1075	347	904	15.8%	0.96 [0.80, 1.15]
Sixten Körper	11	53	17	52	0.9%	0.54 [0.22, 1.30]
V.A. Simonovich	25	228	12	105	1.0%	0.95 [0.46, 1.98]
Yogiraj Ray	10	40	14	40	0.7%	0.62 [0.24, 1.63]
**Subtotal (95% CI)**		**7721**		**7317**	**94.4%**	**0.96 [0.89, 1.03]**
Total events	1911		1870			
Heterogeneity: Chi² = 13.07, df = 11 (P = 0.29); I² = 16%						
Test for overall effect: Z = 1.11 (P = 0.27)						
**16.1.2 >65years**						
CONCOR-1	141	614	63	307	4.3%	1.15 [0.83, 1.61]
Elliott Bennett-Guerrero	16	59	5	15	0.4%	0.74 [0.22, 2.51]
Ling Li	8	52	12	51	0.7%	0.59 [0.22, 1.60]
Romina Libster	2	80	4	80	0.3%	0.49 [0.09, 2.74]
**Subtotal (95% CI)**		**805**		**453**	**5.6%**	**1.03 [0.76, 1.39]**
Total events	167		84			
Heterogeneity: Chi² = 2.65, df = 3 (P = 0.45); I² = 0%						
Test for overall effect: Z = 0.18 (P = 0.86)						
**Total (95% CI)**		**8526**		**7770**	**100.0%**	**0.96 [0.90, 1.03]**
Total events	2078		1954			
Heterogeneity: Chi² = 15.91, df = 15 (P = 0.39); I² = 6%						
Test for overall effect: Z = 1.04 (P = 0.30)						
Test for subgroup differences: Chi² = 0.19, df = 1 (P = 0.66), I² = 0%						

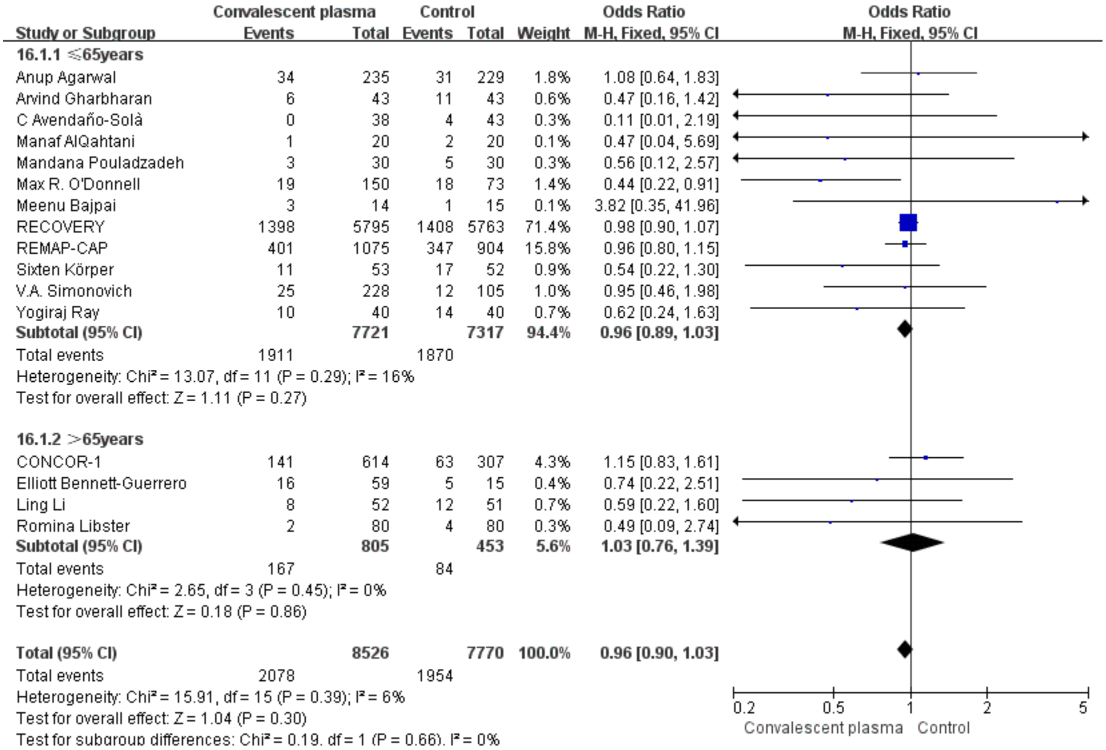

Weights are from fixed-effects analysis. Abbreviation: CI, confidence interval;

Figure 3E Sub-analysis of pooled odds ratios (ORs) in mortality according to volume of infusion(500ml)

	Convalescent plasma		Control			Odds Ratio
Study or Subgroup	Events	Total	Events	Total	Weight	M-H, Fixed, 95% CI
**9.1.1 ≥500ml**						
CONCOR-1	141	614	63	307	4.3%	1.15 [0.83, 1.61]
Mandana Pouladzadeh	3	30	5	30	0.3%	0.56 [0.12, 2.57]
Meenu Bajpai	3	14	1	15	0.1%	3.82 [0.35, 41.96]
REMAP-CAP	401	1075	347	904	15.8%	0.96 [0.80, 1.15]
Sixten Körper	11	53	17	52	0.9%	0.54 [0.22, 1.30]
**Subtotal (95% CI)**		**1786**		**1308**	**21.3%**	**0.98 [0.84, 1.14]**
Total events	559		433			
Heterogeneity: Chi² = 4.53, df = 4 (P = 0.34); I² = 12%						
Test for overall effect: Z = 0.27 (P = 0.79)						
**9.1.2 <500ml**						
Anup Agarwal	34	235	31	229	1.8%	1.08 [0.64, 1.83]
C Avendaño-Solà	0	38	4	43	0.3%	0.11 [0.01, 2.19]
Elliott Bennett-Guerrero	16	59	5	15	0.4%	0.74 [0.22, 2.51]
Ling Li	8	52	12	51	0.7%	0.59 [0.22, 1.60]
Manaf AlQahtani	1	20	2	20	0.1%	0.47 [0.04, 5.69]
RECOVERY	1398	5795	1408	5763	71.4%	0.98 [0.90, 1.07]
Romina Libster	2	80	4	80	0.3%	0.49 [0.09, 2.74]
Yogiraj Ray	10	40	14	40	0.7%	0.62 [0.24, 1.63]
**Subtotal (95% CI)**		**6319**		**6241**	**75.7%**	**0.97 [0.89, 1.06]**
Total events	1469		1480			
Heterogeneity: Chi² = 5.17, df = 7 (P = 0.64); I² = 0%						
Test for overall effect: Z = 0.68 (P = 0.50)						
**9.1.3 Unclear**						
Arvind Gharbharan	6	43	11	43	0.6%	0.47 [0.16, 1.42]
Max R. O'Donnell	19	150	18	73	1.4%	0.44 [0.22, 0.91]
V.A. Simonovich	25	228	12	105	1.0%	0.95 [0.46, 1.98]
**Subtotal (95% CI)**		**421**		**221**	**3.0%**	**0.61 [0.39, 0.97]**
Total events	50		41			
Heterogeneity: Chi² = 2.41, df = 2 (P = 0.30); I² = 17%						
Test for overall effect: Z = 2.09 (P = 0.04)						
**Total (95% CI)**		**8526**		**7770**	**100.0%**	**0.96 [0.90, 1.03]**
Total events	2078		1954			
Heterogeneity: Chi² = 15.91, df = 15 (P = 0.39); I² = 6%						
Test for overall effect: Z = 1.04 (P = 0.30)						
Test for subgroup differences: Chi² = 3.81, df = 2 (P = 0.15), I² = 47.5%						

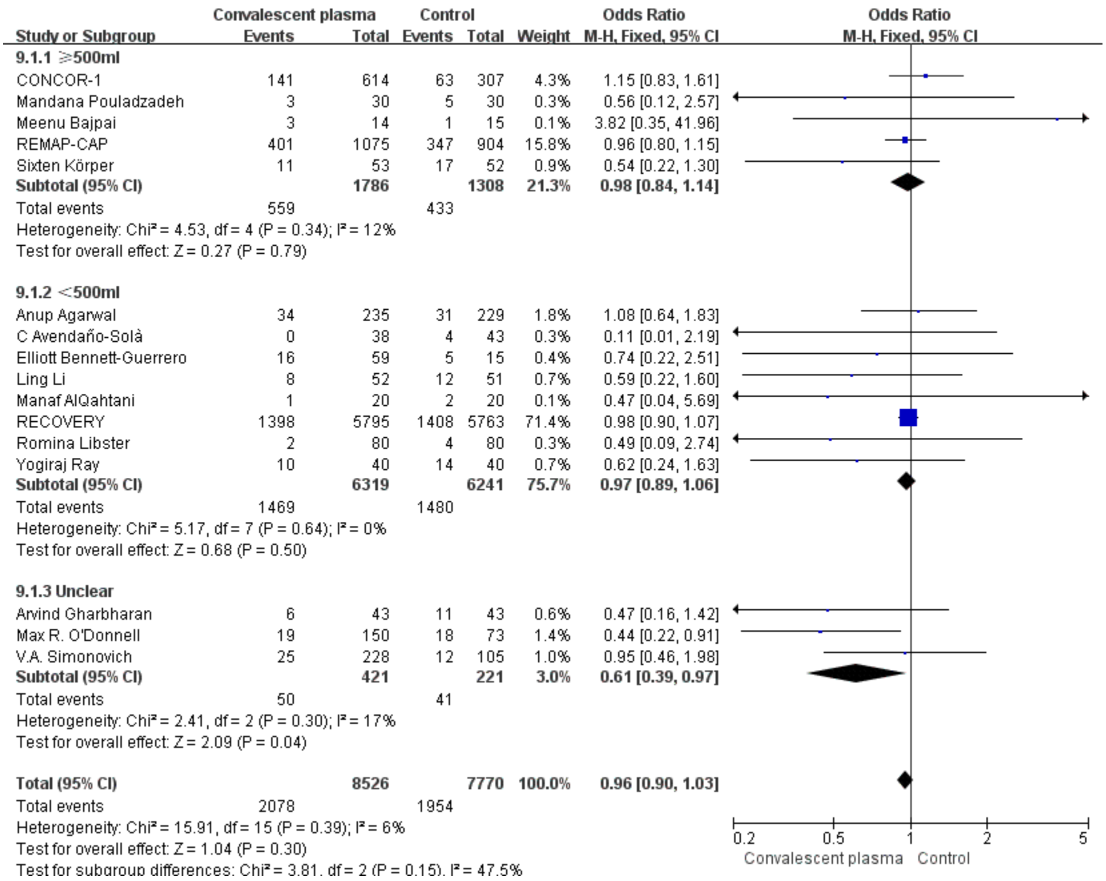

Weights are from fixed-effects analysis. Abbreviation: CI, confidence interval;

Figure 3F Sub-analysis of pooled odds ratios (ORs) in mortality according to volume of infusion(300ml)

Study or Subgroup	Convalescent plasma Events	Convalescent plasma Total	Control Events	Control Total	Weight	Odds Ratio M-H, Fixed, 95% CI
**10.1.1 >300ml**						
Anup Agarwal	34	235	31	229	1.8%	1.08 [0.64, 1.83]
CONCOR-1	141	614	63	307	4.3%	1.15 [0.83, 1.61]
Elliott Bennett-Guerrero	16	59	5	15	0.4%	0.74 [0.22, 2.51]
Manaf AlQahtani	1	20	2	20	0.1%	0.47 [0.04, 5.69]
Mandana Pouladzadeh	3	30	5	30	0.3%	0.56 [0.12, 2.57]
Meenu Bajpai	3	14	1	15	0.1%	3.82 [0.35, 41.96]
REMAP-CAP	401	1075	347	904	15.8%	0.96 [0.80, 1.15]
Sixten Körper	11	53	17	52	0.9%	0.54 [0.22, 1.30]
Yogiraj Ray	10	40	14	40	0.7%	0.62 [0.24, 1.63]
**Subtotal (95% CI)**		**2140**		**1612**	**24.3%**	**0.97 [0.84, 1.12]**
Total events	620		485			
Heterogeneity: Chi² = 6.03, df = 8 (P = 0.64); I² = 0%						
Test for overall effect: Z = 0.41 (P = 0.68)						
**10.1.2 <300ml**						
C Avendaño-Solà	0	38	4	43	0.3%	0.11 [0.01, 2.19]
Ling Li	8	52	12	51	0.7%	0.59 [0.22, 1.60]
RECOVERY	1398	5795	1408	5763	71.4%	0.98 [0.90, 1.07]
Romina Libster	2	80	4	80	0.3%	0.49 [0.09, 2.74]
**Subtotal (95% CI)**		**5965**		**5937**	**72.6%**	**0.97 [0.90, 1.06]**
Total events	1408		1428			
Heterogeneity: Chi² = 3.66, df = 3 (P = 0.30); I² = 18%						
Test for overall effect: Z = 0.60 (P = 0.55)						
**10.1.3 Unclear**						
Arvind Gharbharan	6	43	11	43	0.6%	0.47 [0.16, 1.42]
Max R. O'Donnell	19	150	18	73	1.4%	0.44 [0.22, 0.91]
V.A. Simonovich	25	228	12	105	1.0%	0.95 [0.46, 1.98]
**Subtotal (95% CI)**		**421**		**221**	**3.0%**	**0.61 [0.39, 0.97]**
Total events	50		41			
Heterogeneity: Chi² = 2.41, df = 2 (P = 0.30); I² = 17%						
Test for overall effect: Z = 2.09 (P = 0.04)						
**Total (95% CI)**		**8526**		**7770**	**100.0%**	**0.96 [0.90, 1.03]**
Total events	2078		1954			
Heterogeneity: Chi² = 15.91, df = 15 (P = 0.39); I² = 6%						
Test for overall effect: Z = 1.04 (P = 0.30)						
Test for subgroup differences: Chi² = 3.81, df = 2 (P = 0.15), I² = 47.5%						

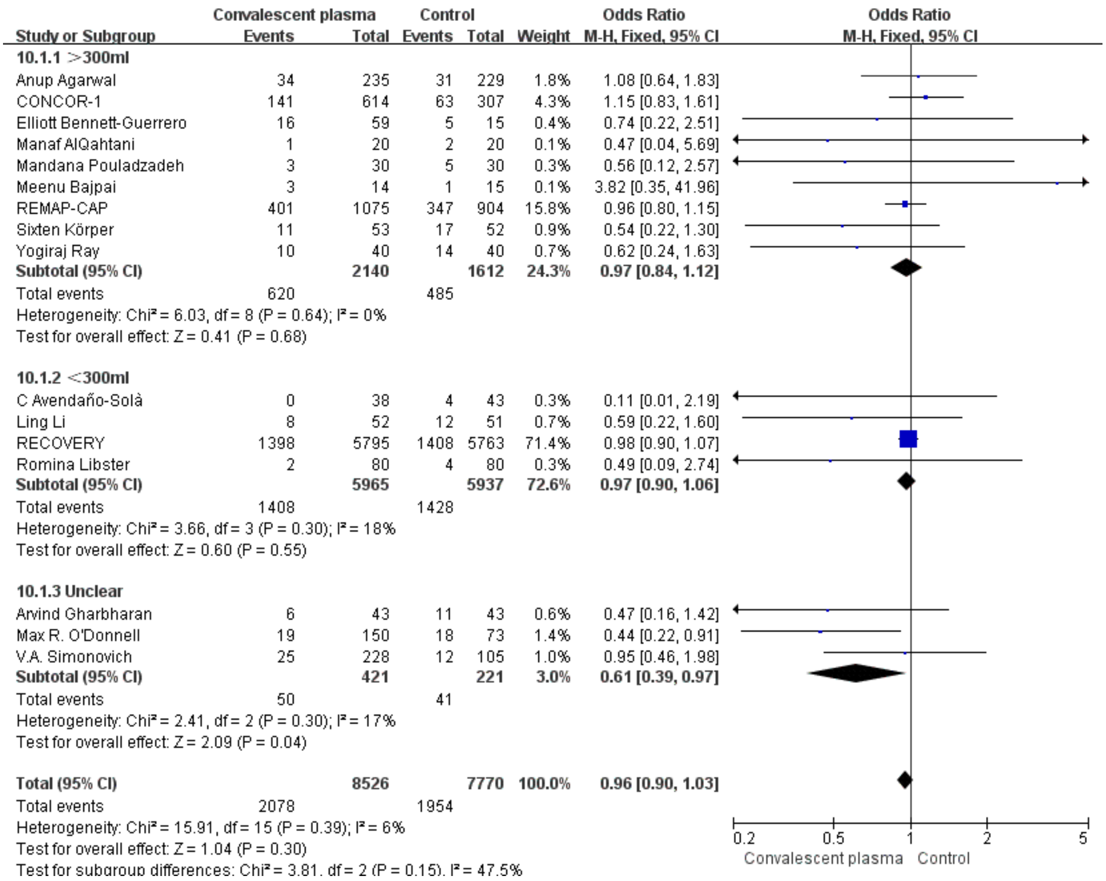

Weights are from fixed-effects analysis. Abbreviation: CI, confidence interval;

Figure 3G the funnel plot of mortality

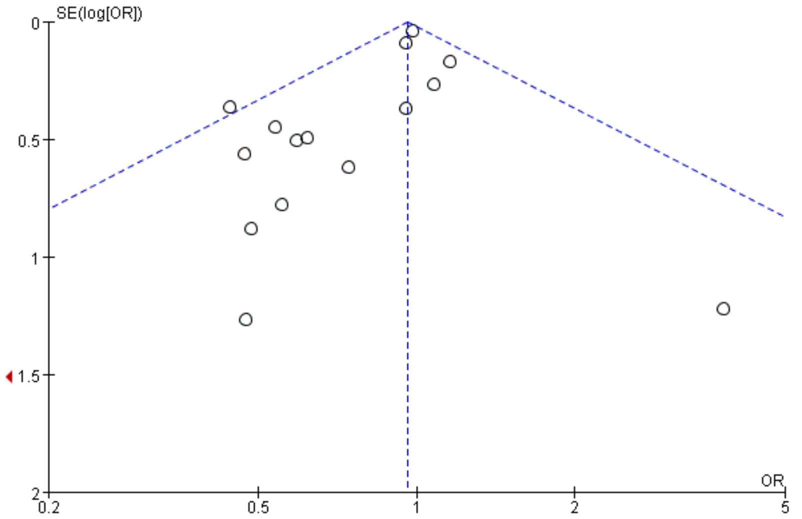

Figure 3H the Egger test in mortality

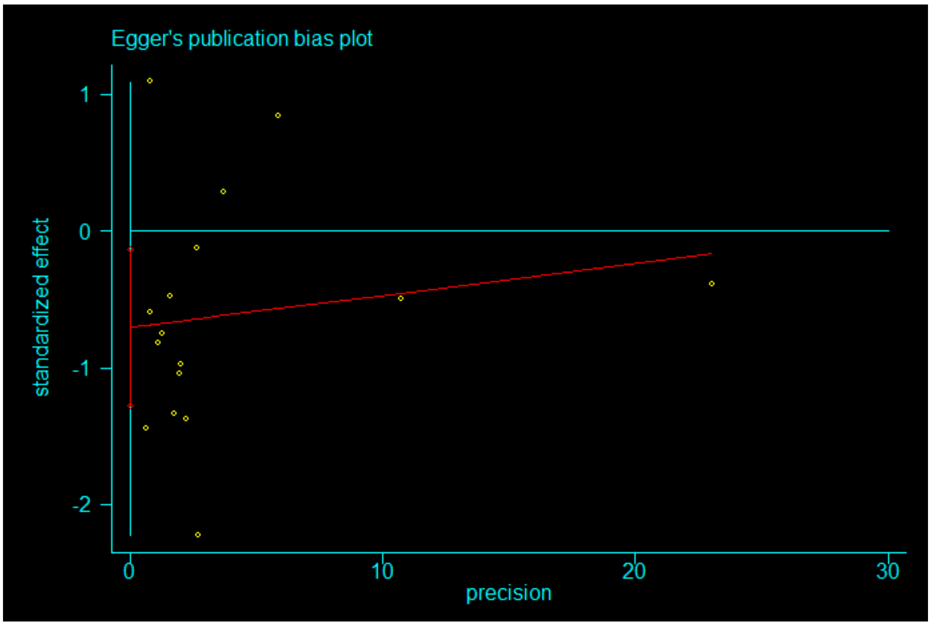

Figure 4B   the funnel plot of mortality of 28-day

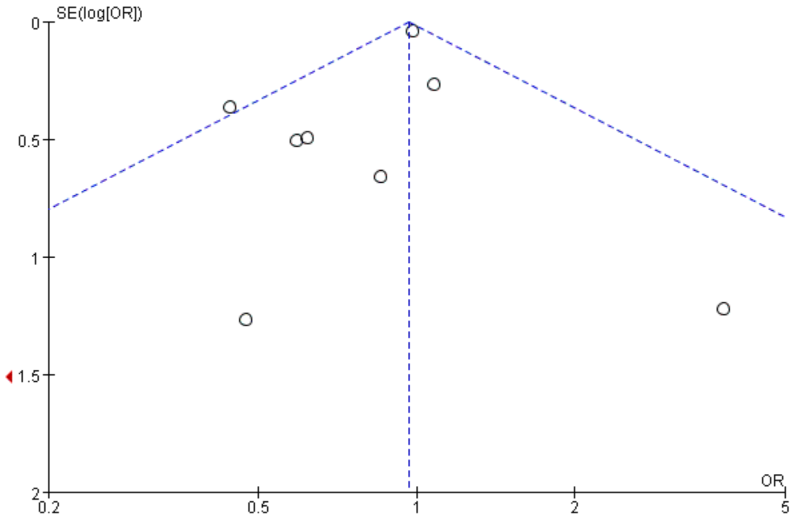

Figure 4C   the Egger test in mortality of 28 day

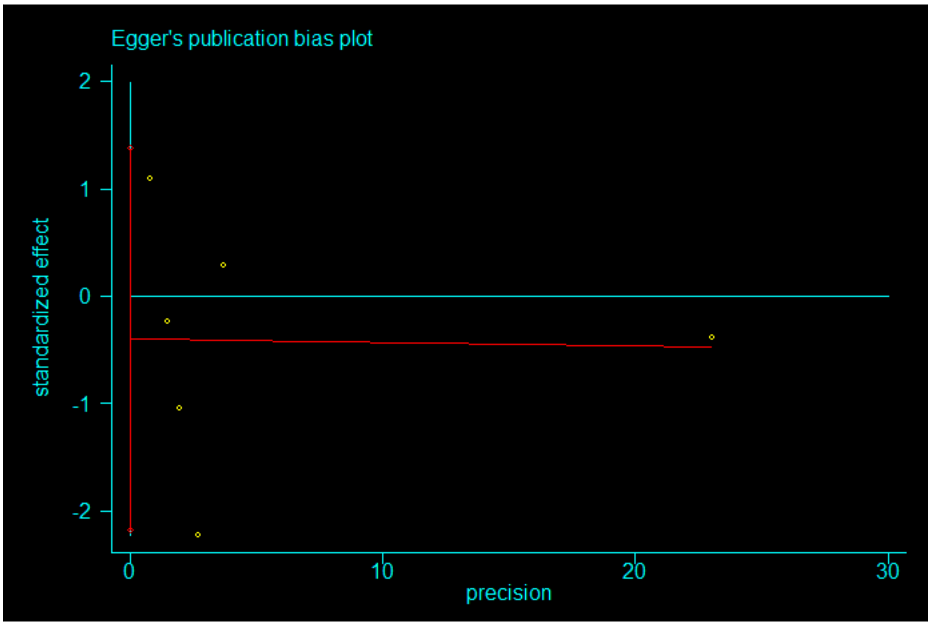

Figure 5A the funnel plot of hospital stay

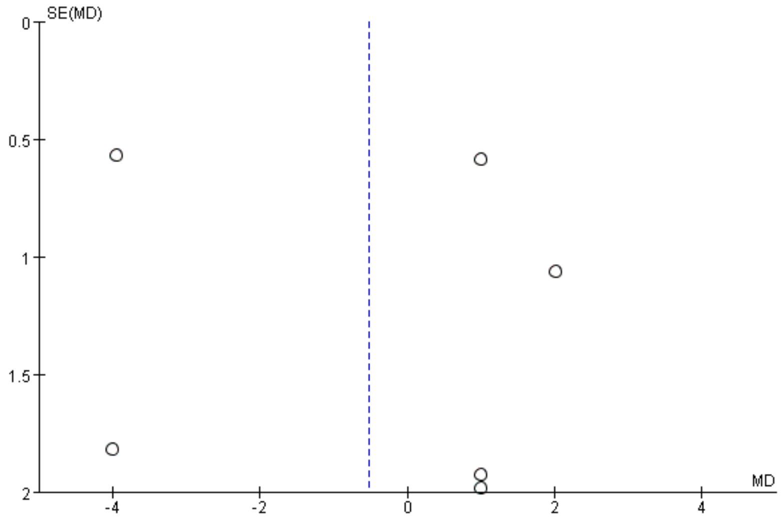

Figure 5B the Egger test in hospital stay

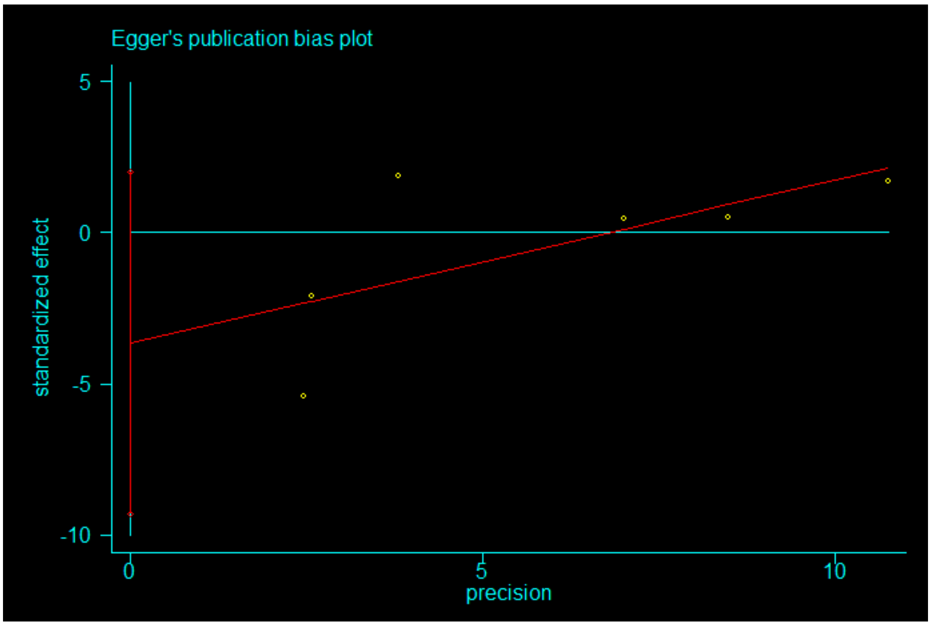

Figure 6A the funnel plot of the duration of ICU

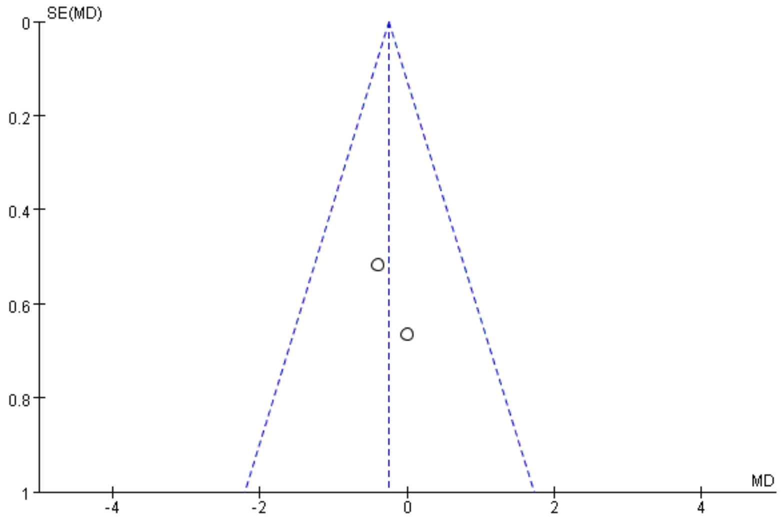

Figure 6B the Egger test in the duration of ICU

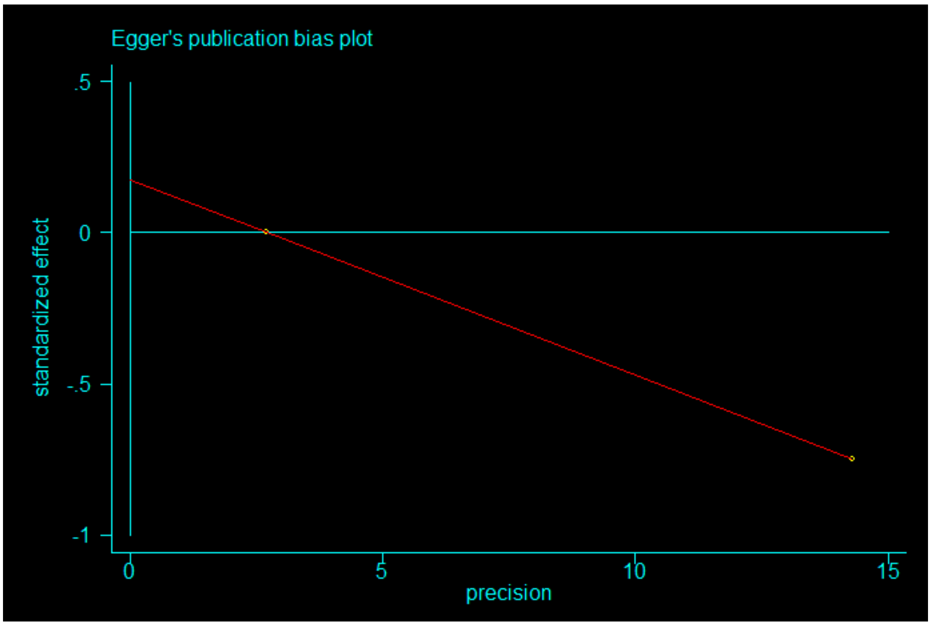

Figure 7A the funnel plot of the duration of MV

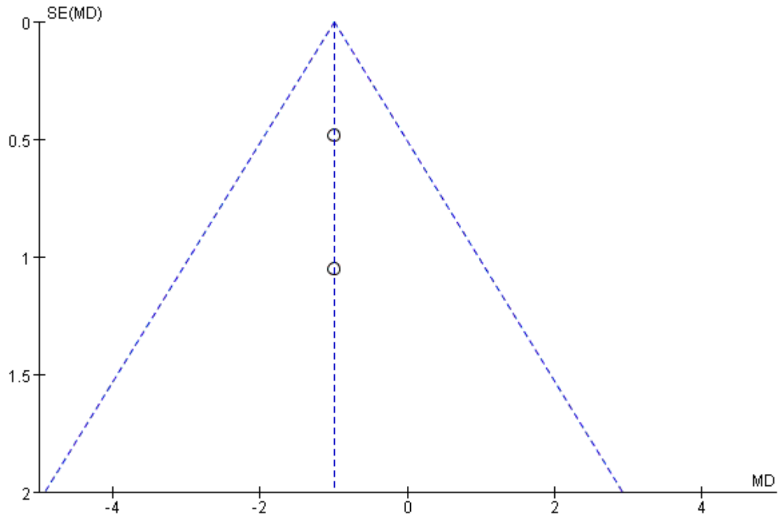

Figure 7B the Egger test in the duration of MV

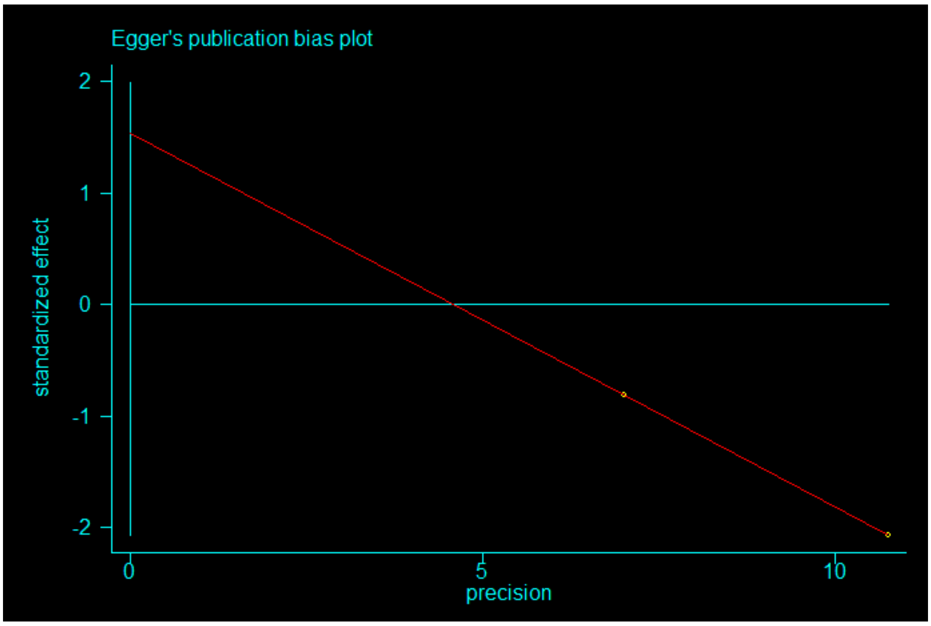

Figure 8A the funnel plot of the rate of ICU

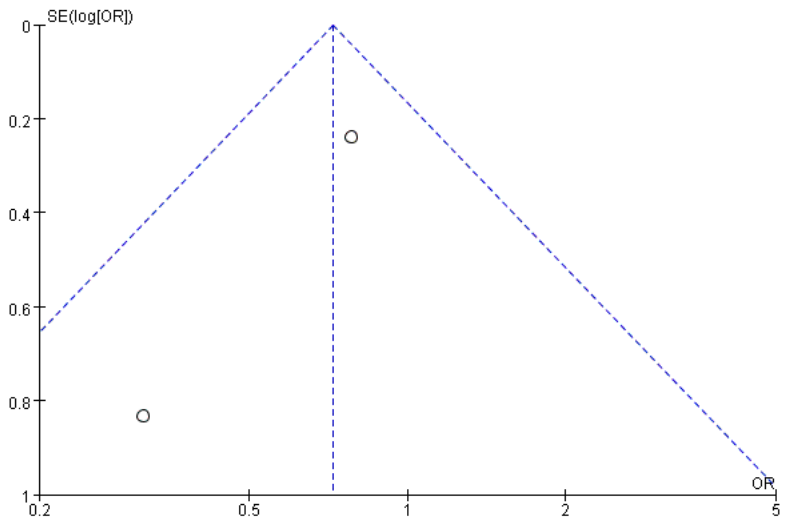

Figure 8B the Egger test in the rate of ICU

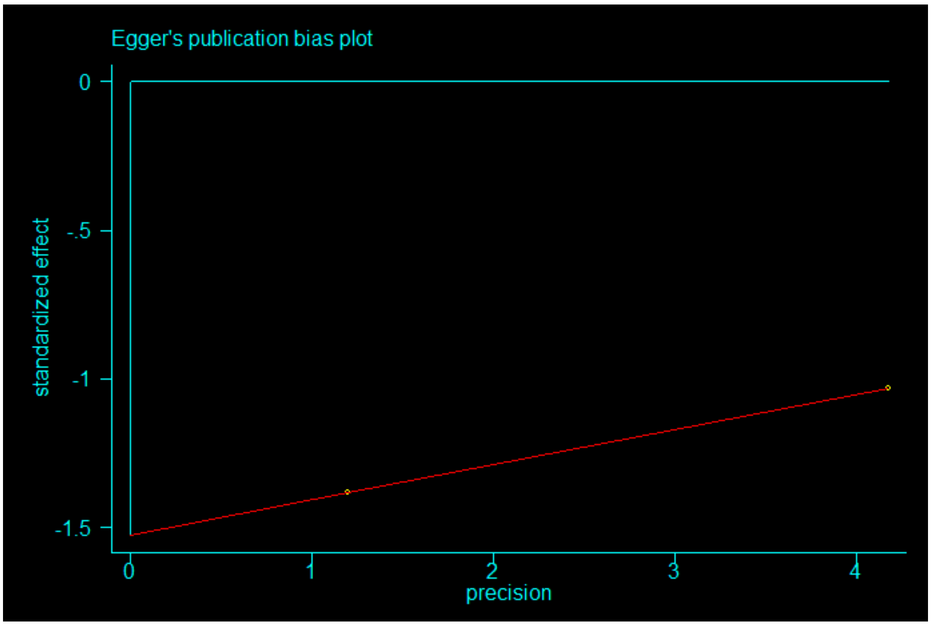

Figure 9A the funnel plot of the rate of mechanic ventilation

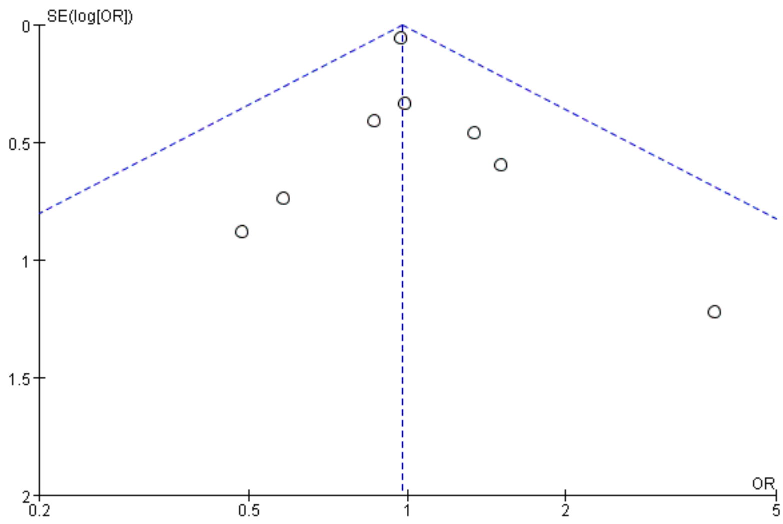

Figure 9B the Egger test in the rate of mechanic ventilation

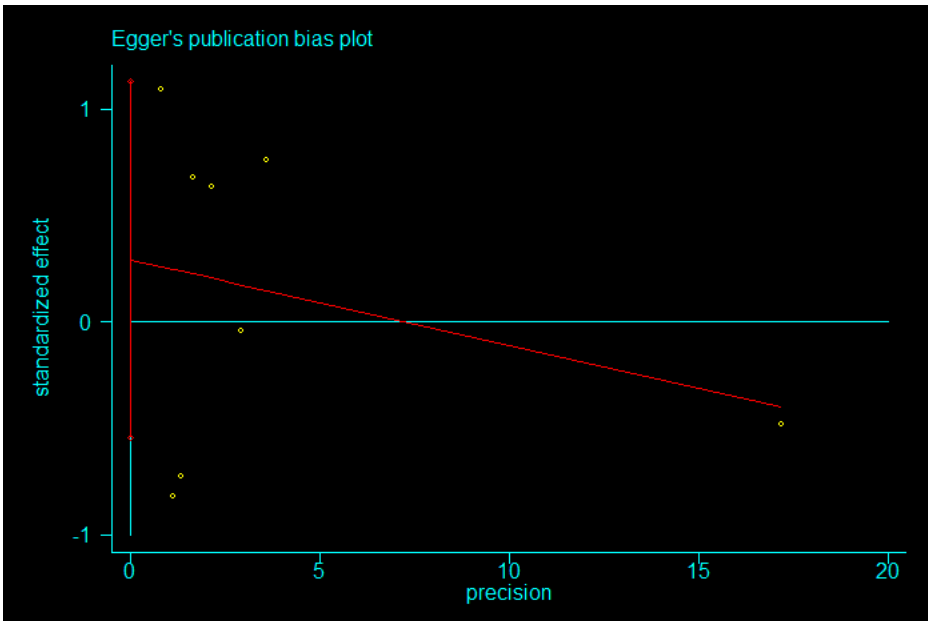

Supplement: Supplementary file 1 [file Data_Sheet_1.pdf]
